# Correction: The genetic consequences of dog breed formation—Accumulation of deleterious genetic variation and fixation of mutations associated with myxomatous mitral valve disease in cavalier King Charles spaniels

**DOI:** 10.1371/journal.pgen.1010039

**Published:** 2022-01-27

**Authors:** Erik Axelsson, Ingrid Ljungvall, Priyasma Bhoumik, Laura Bas Conn, Eva Muren, Åsa Ohlsson, Lisbeth Høier Olsen, Karolina Engdahl, Ragnvi Hagman, Jeanette Hanson, Dmytro Kryvokhyzha, Mats Pettersson, Olivier Grenet, Jonathan Moggs, Alberto Del Rio-Espinola, Christian Epe, Bruce Taillon, Nilesh Tawari, Shrinivas Mane, Troy Hawkins, Åke Hedhammar, Philippe Gruet, Jens Häggström, Kerstin Lindblad-Toh

## Notice of republication

An incorrect version of S1 Text was published in error. This article was republished on January 13, 2022 to correct for this error. Please download this article again to view the correct version.

## References

[1] Axelsson E, Ljungvall I, Bhoumik P, Conn LB, Muren E, Ohlsson Å, et al. (2021) The genetic consequences of dog breed formation—Accumulation of deleterious genetic variation and fixation of mutations associated with myxomatous mitral valve disease in cavalier King Charles spaniels. PLoS Genet 17(9): e1009726. 10.1371/journal.pgen.1009726 34473707 PMC8412370

