# Supplementary material for: The genetic consequences of dog breed formation—Accumulation of deleterious genetic variation and fixation of mutations associated with myxomatous mitral valve disease in cavalier King Charles spaniels
Source: PLoS Genet. 2021 Sep 2;17(9):e1009726. doi: 10.1371/journal.pgen.1009726 (PMC8412370; doi:10.1371/journal.pgen.1009726)
Supplement: S1 Text — This file contains all supporting figures and tables referred to in the manuscript. (DOCX) [file pgen.1009726.s001.docx]

**Supporting information**

**Fig 1. Single nucleotide variant detection power.** Power of the SNV calling was estimated by comparing variant calls from our whole genome resequencing data with those based on array genotyping (HD 170 canine array) in the same set of 20 cKCs individuals sequenced here (Vaysse et al., 2011). Power was calculated as the fraction of biallelic variable sites detected using array genotyping that was detected using whole genome resequencing.

**Fig 2. Cumulative proportion of single nucleotide variants detected.** Cumulative proportion of all chromosome 1 and 2 variants identified in a particular breed that were discovered in a single genome and with every additional individual analyzed.

**Fig 3. Neighbour-Joining tree showing the relatedness among the breeds analysed.**

The pair-wise distance matrix for the tree is based on calculations of genome-wide F_ST_ between breed pairs.

**Fig 4. Nucleotide diversity and allele frequency distribution in the eight breeds analysed.** The number of variants per megabase (Mb) at different derived allele counts divided by expectations under the neutral coalescent (*1/i* where *i* is the derived allele count). This represents an estimate of theta per Mb and shows a clear lack of rare ancestral alleles, but excess of derived nearly fixed alleles, in dog breeds. A drop in theta at a derived allele count of 20 lacks biological significance and is caused by the fact that alleles often were called in 39 out of the 40 chromosomes sequenced per breed, and rescaling allele counts in these individuals resulted in an allele count of 21 (i.e. (20/39)*40=20.51).

**Fig 5. Demographic history of the eight breeds.** Model free inferences of demographic histories were estimated based on breed specific site frequency spectra using the software *epos*. Thick line shows median estimate and thin lines indicate 2.5 and 97.5 percentiles, respectively, based on 1000 bootstrap replicates.

**Fig 6. Decay of linkage disequilibrium (LD) in the eight dog breeds.** Mean LD, as measured using r^2^, between pairs of SNVs at different distances (measured in kilobases (Kb)) from each other.

**Fig 7. Length distribution of runs of homozygosity in the eight dog breeds analysed.** The average number of runs of homozygosity (ROH) per individual in each breed plotted for different ROH lengths as measured in megabases (Mb).

**Fig 8. Relationship between age at screening, genotype and disease status in the Swedish dachshund population.** Box plot displaying the distribution of age at screening (on the Y-axis) for different genotypes at the 9 segregating candidate variants (on the X-axis) in Swedish dachshunds characterized as healthy controls (Normal, colored in blue) or as affected by myxomatous mitral valve disease (MMVD, colored in red), respectively. A bold horizontal line represents the median age at screening. Boxes include the 25th and 75th percentiles, respectively and whiskers extend a further 1.5 * IQR (interquartile range) from the box. Circles mark outliers. The number of sampled individuals for each category is displayed on top of each box.

**Fig 9. Linkage disequilibrium between the *NEBL* candidate variants in the Swedish dachshund population. a)** Pairwise D’ values are shown unless D’=1. Red color indicates high D’, white indicates low D’ and blue reflects pairs of variants that have high D’ but low LOD score. **b)** Pairwise r^2^ values are displayed. Dark grey color indicates high r^2^, lighter grey or white indicates low r^2^.

**Fig 10. Results of electrophoretic mobility shift assays.** Results of 10 representative electro mobility shift assays (EMSAs) testing the ability of candidate variants *NEBL 1*, *NEBL 2*, *NEBL 3* and *NEBL 4* to bind nuclear protein extract from rat cardiomyocytes (represented by H9C2 cells), rat heart valve interstitial cells (represented by RAVIC cells) and canine kidney cells (represented by MDCK cells). Each assay is enclosed by a square, within which three lanes show results of tests using probes with reference allele (REF), and three lanes show results for tests using probes with alternative allele (ALT), respectively. The following experimental conditions apply to the three lanes: lane 1 – labeled probe, no protein extract; lane 2 – labeled probe + protein extract; lane 3 – labeled probe + protein extract + excess of unlabeled probe to outcompete binding to labeled probe. The three leftmost lanes residing outside of any square depicts results of EBNA controls. Lanes marked *eM*, *eH* and *eR* contain only cell extract from MDCK, H9C2 and RAVIC cells, respectively. Experimental ids in each panel refer to detailed inhouse experimental documentation. Description of variants and cell line combination tested in each figure panel: **a)** *NEBL 1* in MDCK and RAVIC; **b)** *NEBL 1* in MDCK and H9C2; **c)** Results are displayed for four different oligos tested for binding to cell extract from MDCK: *NEBL 1 + NEBL 1.2 mutation* (ref frame) - oligo has alternative alleles at *NEBL 1* and *NEBL 1.2*.); *only NEBL 1.2 mutation* (purple frame) - oligo has reference allele at *NEBL 1*, but alternative allele at *NEBL 1.2*; *no mutation* (green frame) – oligo has reference allele at *NEBL 1* and *NEBL 1.2*; *NEBL 1 (*yellow frame*)* - oligo has alternative allele at *NEBL 1*; **d)** Replicate one of *NEBL 2* in MDCK and H9C2; **e)** Replicate two of *NEBL 2* in MDCK and H9C2; **f)** *NEBL 2* in RAVIC; **g)** *NEBL 3* in MDCK and H9C2; **h)** *NEBL 3* in RAVIC; **i)** *NEBL 4* in H9C2 and RAVIC; and **j)** *NEBL 4* in MDCK.

**Table 1.** **Power and accuracy of the SNV calling and genotyping.** Total number of SNVs and INDELs discovered before (*all*) and after filtering (*filtered*) to keep 99.9% of the SNVs and 99.0% of the INDELs in the truth sets. Power and accuracy of the SNV calling and genotyping was estimated by comparing variant calls from our whole genome resequencing data with those based on array genotyping (HD 170 canine array) in the same set of 20 CKCS individuals sequenced here (Vaysse et al., 2011). *SNV detection power* was calculated as the fraction of biallelic variable sites detected using array genotyping that was detected using whole genome resequencing (WGS). *FDR* refers to the fraction of biallelic variable sites detected using WGS that were also interrogated using array genotyping, but found not to be segregating based on that technology. Similarly, genotype accuracy (*GT accuracy*) was estimated as the fraction of overlapping WGS calls showing identical genotypes in the array data. *Missingness* refers to the number of individual genotypes not called at sites that were detected using WGS. *Total missingness* also adds missing individual genotype calls at sites that were found to be segregating using array genotyping but not using WGS.

**Table 2. Genetic diversity within and differentiation between breeds analysed.** Average heterozygosity per breed was calculated for all sites found to be segregating in the complete data set. Differentiation in one breed relative to all other breeds was calculated as the mean of all pair-wise F_ST_ estimates.

**Table 3. Relative abundance of derived potentially deleterious alleles, R_A/B_, at synonymous sites for all pairwise breed comparisons.** Three numbers are presented in each square. Top number – R_A/B_ values, refer to relative abundance of potential deleterious alleles in the breed to the right of the row relative to in breed on top of the column. A value below 1 indicates that there are more deleterious alleles in the breed on top of the column relative to in the breed to the right of the row and vice versa. Middle number – 95% confidence interval. Bottom number – p-value. Color highlighting refers to R_A/B_ values that deviate significantly from 1, after correction for multiple testing (orange), or only without correction for multiple testing (yellow), respectively.

**Table 4. Relative abundance of derived potentially deleterious alleles, R_A/B_, at nonsynonymous sites for all pairwise breed comparisons.** Three numbers are presented in each square. Top number – R_A/B_ values, refer to relative abundance of potential deleterious alleles in the breed to the right of the row relative to in breed on top of the column. A value below 1 indicates that there are more deleterious alleles in the breed on top of the column relative to in the breed to the right of the row and vice versa. Middle number – 95% confidence interval. Bottom number – p-value. Color highlighting refers to R_A/B_ values that deviate significantly from 1, after correction for multiple testing (orange), or only without correction for multiple testing (yellow), respectively.

**Table 5. Relative abundance of derived potentially deleterious alleles, R_A/B_, at loss of function (LoF) sites for all pairwise breed comparisons.** *LoF* included variants affecting sites characterized as belonging to one of the following SNPeff categories: *CODON_CHANGE_PLUS_CODON_DELETION', 'CODON_CHANGE_PLUS_CODON_INSERTION', 'CODON_DELETION', 'CODON_INSERTION', 'EXON_DELETED', 'FRAME_SHIFT', 'SPLICE_SITE_ACCEPTOR', 'SPLICE_SITE_DONOR', 'START_LOST', 'STOP_GAINED'.* Three numbers are presented in each square. Top number – R_A/B_ values, refer to relative abundance of potential deleterious alleles in the breed to the right of the row relative to in breed on top of the column. A value below 1 indicates that there are more deleterious alleles in the breed on top of the column relative to in the breed to the right of the row and vice versa. Middle number – 95% confidence interval. Bottom number – p-value. Color highlighting refers to R_A/B_ values that deviate significantly from 1, after correction for multiple testing (orange), or only without correction for multiple testing (yellow), respectively.

**Table 6. Relative abundance of derived potentially deleterious alleles, R_A/B_, at moderately conserved sites (2>PhyloP_100 vertebrates_<=5) for all pairwise breed comparisons.** Three numbers are presented in each square. Top number – R_A/B_ values, refer to relative abundance of potential deleterious alleles in the breed to the right of the row relative to in breed on top of the column. A value below 1 indicates that there are more deleterious alleles in the breed on top of the column relative to in the breed to the right of the row and vice versa. Middle number – 95% confidence interval. Bottom number – p-value. Color highlighting refers to R_A/B_ values that deviate significantly from 1, after correction for multiple testing (orange), or only without correction for multiple testing (yellow), respectively.

**Table 7. Link between levels of recessive, but not additive load, and levels of genetic diversity.** Relative abundance of derived potentially deleterious alleles, R_A/B_, and derived potentially deleterious alleles in a homozygous state, R^2^_A/B_, at synonymous (S), nonsynonymous (NS), moderately conserved sites (2<PhyloP_100 vertebrates_>=5) and highly conserved sites (PhyloP_100 vertebrates_>5) for all pairwise breed comparisons involving cKCs (cavalier King Charles spaniel). Top number – R_A/B_ (or R^2^_A/B_), refers to relative abundance of potential deleterious alleles in cKCs relative to in breed indicated at start of row. Middle number – 95% confidence interval. Bottom number – p-value. Color highlighting refers to R_A/B_ values that deviate significantly from 1, after correction for multiple testing (orange with asterix), or only without correction for multiple testing (yellow). *Avg. het* is the average genome wide heterozygosity per breed. *Seg. sites* is the total number of segregating sites per breed. Bottom row in table displays results of correlation test between R_A/B_ (or R^2^_A/B_) and total number of segregating sites per breed in each breed used for comparison with cKCs.

**Table 8. R_A/B_, at highly conserved sites (PhyloP_100 vertebrates_>5) excluding regions under potential recent selection in cKCs.** Three numbers are presented in each square. Top number – R_A/B_ values, refer to relative abundance of potential deleterious alleles in the breed to the right of the row relative to in breed on top of the column. A value below 1 indicates that there are more deleterious alleles in the breed on top of the column relative to in the breed to the right of the row and vice versa. Middle number – 95% confidence interval. Bottom number – p-value. Color highlighting refers to R_A/B_ values that deviate significantly from 1, after correction for multiple testing (orange), or only without correction for multiple testing (yellow), respectively.

**Table 9. Relative abundance of derived potentially deleterious alleles in a homozygous state, R^2^_A/B_, at synonymous sites for all pairwise breed comparisons.** Three numbers are presented in each square. Top number - R^2^_A/B_ values, refer to relative abundance of potential deleterious alleles in a homozygous state in the breed to the right of the row relative to in breed on top of the column. A value below 1 indicates that there are more deleterious alleles in a homozygous state in the breed on top of the column relative to in the breed to the right of the row and vice versa. Middle number – 95% confidence interval. Bottom number – p-value. Color highlighting refers to R_A/B_ values that deviate significantly from 1, after correction for multiple testing (orange and asterisk), or only without correction for multiple testing (yellow), respectively.

**Table 10. Relative abundance of derived potentially deleterious alleles in a homozygous state, R^2^_A/B_, at nonsynonymous sites for all pairwise breed comparisons.** Three numbers are presented in each square. Top number - R^2^_A/B_ values, refer to relative abundance of potential deleterious alleles in a homozygous state in the breed to the right of the row relative to in breed on top of the column. A value below 1 indicates that there are more deleterious alleles in a homozygous state in the breed on top of the column relative to in the breed to the right of the row and vice versa. Middle number – 95% confidence interval. Bottom number – p-value. Color highlighting refers to R_A/B_ values that deviate significantly from 1, after correction for multiple testing (orange and asterix), or only without correction for multiple testing (yellow), respectively.

**Table 11. Relative abundance of derived potentially deleterious alleles in a homozygous state, R^2^_A/B_, at loss of function sites (*LoF)* for all pairwise breed comparisons.** *LoF* included variants affecting sites characterized as belonging to one of the following SNPeff categories: *CODON_CHANGE_PLUS_CODON_DELETION', 'CODON_CHANGE_PLUS_CODON_INSERTION', 'CODON_DELETION', 'CODON_INSERTION', 'EXON_DELETED', 'FRAME_SHIFT', 'SPLICE_SITE_ACCEPTOR', 'SPLICE_SITE_DONOR', 'START_LOST', 'STOP_GAINED'.* Three numbers are presented in each square. Top number - R^2^_A/B_ values, refer to relative abundance of potential deleterious alleles in a homozygous state in the breed to the right of the row relative to in breed on top of the column. A value below 1 indicates that there are more deleterious alleles in a homozygous state in the breed on top of the column relative to in the breed to the right of the row and vice versa. Middle number – 95% confidence interval. Bottom number – p-value. Color highlighting refers to R_A/B_ values that deviate significantly from 1, after correction for multiple testing (orange and asterix), or only without correction for multiple testing (yellow), respectively.

**Table 12. Relative abundance of derived potentially deleterious alleles in a homozygous state, R^2^_A/B_, at moderately conserved sites (2>Phylop_100 vertebrates_<=5) for all pairwise breed comparisons.** Three numbers are presented in each square. Top number - R^2^_A/B_ values, refer to relative abundance of potential deleterious alleles in a homozygous state in the breed to the right of the row relative to in breed on top of the column. A value below 1 indicates that there are more deleterious alleles in a homozygous state in the breed on top of the column relative to in the breed to the right of the row and vice versa. Middle number – 95% confidence interval. Bottom number – p-value. Color highlighting refers to R_A/B_ values that deviate significantly from 1, after correction for multiple testing (orange and asterix), or only without correction for multiple testing (yellow), respectively.

**Table 13. Relative abundance of derived potentially deleterious alleles in a homozygous state, R^2^_A/B_, at highly conserved sites (Phylop_100 vertebrates_>5) for all pairwise breed comparisons.** Three numbers are presented in each square. Top number - R^2^_A/B_ values, refer to relative abundance of potential deleterious alleles in a homozygous state in the breed to the right of the row relative to in breed on top of the column. A value below 1 indicates that there are more deleterious alleles in a homozygous state in the breed on top of the column relative to in the breed to the right of the row and vice versa. Middle number – 95% confidence interval. Bottom number – p-value. Color highlighting refers to R_A/B_ values that deviate significantly from 1, after correction for multiple testing (orange and asterix), or only without correction for multiple testing (yellow), respectively.

**Table 14. High frequency derived variants per breed.** Number of high frequency

(F_ST_ or V_ST_ >= 0.7) derived SNVs, INDELs, large deletions and CNVs found per breed at all sites and at conserved sites (conserved SNVs and INDELs defined as having Phylop_100 vertebrates_<=2 or Phylop_40 mammals_<=2; conserved large detetions and CNVs defined as including sequence with phastcon scores<0) , respectively. Number of sites fixed for derived allele per breed is also reported.

**Table 15. High frequency derived variants with known phenotypic effect.** *PhyloP score* refers to scores estimated using 100 vertebrates. *Average pairwise F_ST_ rank* refers to the rank of the indicated variant’s F_ST_ among all SNVs and INDELs detected in the breed indicated under *Breed with high F_ST_*. *Total F_ST_/V_ST_* is calculated for *SNVs* and *INDELs*, and *large deletions* and *CNVs*, respectively, across all 8 breeds and *Total F_ST_/V_ST_ rank* refers to the rank of the indicated variant’s total F_ST_ and total V_ST_ among all variants detected. High ranking *Total F_ST_ and V_ST_* values primarily identify variants for which highly differentiated alleles are found in groups of two or more breeds, as opposed to a high *Average pairwise F_ST_ rank* which primarily identifies sites for which a derived allele has reached high frequency in a single breed. *Status* (causative, likely causative or potentially causative): indicates the strength of the link between the mutation and the phenotypic evidence). *Type*: type of mutation. *Gene*: gene affected by mutation. *Phenotype*: phenotype linked to mutation.

**Table 16. Candidate MMVD risk variants affecting down regulated genes in canine MMVD cases.** Five high frequency derived variants in cKCs that affect moderately or highly conserved sites (PhyloP_100vertebrates_ or PhyloP_46mammals_>=2) located within 5 Kb up- and downstream of genes that were previously shown to be downregulated in canine MMVD cases relative to in healthy controls (Lu et al., 2015). *Ref*: reference genome allele. *Alt*: alternative allele. *Ancestral:* ancestral allele state as inferred in cat and fox reference genome. *Wolf alt AF*: allele frequency of alternative allele in three wolfs (one American wolf, one Iranian wolf and one Chinese wolf). *Gene*: name of gene affected by mutation. *Expr. fold change*: Log_10_-fold expression change in MMVD cases relative to in healthy controls (Lu et al., 2015). *SNPeff*: snpeff annotation of site affected by mutation. *PhyloP 100 vert.* – conservation score for site based on comparison of 100 vertebrates. *PhyloP 46 mamm.* – conservation score for site based on comparison of 46 mammals. *Average pair wise FST* and the *reference allele frequency* is presented for each breed in study (cKCs – cavalier King Charles spaniel, WHwt – West Highland white terrier).

**Table 17.** Additional candidate MMVD risk variants near *NEBL* gene. Three additional candidate variants in CKCS that affect moderately or highly conserved sites (*PhyloP*_100vertebrates_ or *PhyloP*_46mammals_>=2) located within a region spanning 1 Mb centered around the *NEBL* gene (chr2:11,650,000-12,650,000)**.** *Alt*: alternative allele. *Cat*: cat reference genome allele. *Ancestral:* ancestral allele state as inferred in cat and fox reference genome. *Wolf alt AF*: allele frequency of alternative allele in three wolfs (one American wolf, one Iranian wolf and one Chinese wolf). *Gene*: name of gene affected by mutation. *SNPeff*: snpeff annotation of site affected by mutation. *PhyloP 100 vertebrates* – conservation score for site based on comparison of 100 vertebrates. *PhyloP 46 mammals* – conservation score for site based on comparison of 46 mammals. *Average pair wise F_ST_* and the *reference allele frequency* is presented for each breed in study (cKCs – cavalier King Charles spaniel, WHwt – West Highland white terrier).

**Table 18.** **Candidate allele frequencies, MMVD incidence and across breed association analysis to MMVD.** Reference allele frequencies for the 10 candidate MMVD risk variants. *n* – number of dogs genotyped per breed. *Weight* - sex averaged weight for breed. *MMVD incidence* was estimated for Swedish dogs and calculated as the number of reimbursements for MMVD related symptoms per 10,000 years of total insurance time (see Methods for details) in dogs with veterinary care insurance in Agria Pet Insurance. *Heart disease incidence* was estimated in a similar manner but was based on, in addition to MMVD specific symptoms, more general symptoms of heart disease. *P-value*, *intercept*, slope of line (β) and *adjusted R^2^* is presented for linear regressions investigating if 1) average breed weight predicts MMVD incidence, 2) allele frequencies at each of the 10 candidate variants predicts *MMVD incidence* across all dogs, 3) allele frequencies at each of the 10 candidate variants predicts *heart disease incidence* across all dogs, 4) allele frequencies at each of the 10 candidate variants predicts *MMVD incidence* across all dogs after excluding cKCs, 5) allele frequencies at each of the 10 candidate variants predicts *heart disease incidence* across all dogs after excluding cKCs and 6) *allele frequencies* at each of the 10 candidate variants together with *weight* predicts *MMVD incidence* across all dogs after excluding cKCs. Significant regression models are marked in bold.

**Table 19. Age, sex and candidate allele frequencies in the Swedish dachshund case control sample.** Dogs sampled (*n*), number of males and females (*sex f/m*), average age (*age*) and allele frequencies (stated beneath each candidate variant id, i.e. *NEBL 1, NEBL 2, NEBL 3, NEBL 4, NEBL 5, NEBL 6, LPHN2, HDGFL1, HTR1F*) for each of the 9 candidate variants found to be segregating in the Swedish dachshund MMVD case control population. *P-value*, slope of line (β) and adjusted R^2^ (*R^2^*) is presented for univariate linear regressions investigating if *age*, *sex* or candidate variant genotype can predict MMVD disease status (*status*: 0=healthy, 1=case) or MMVD graded disease status (*graded_status*: 0=healthy, 1=mild symptoms, 2=moderate symptoms, 3=severe symptoms).

**Table 20. Relationship between age, sex and candidate allele frequencies at candidate variants in Swedish dachshund population.** *Intercept* and adjusted R^2^ (*R^2^*) for multivariate association analyses investigating if *i)* *age* and candidate variant genotype (*gt*) or *ii) age, sex* and candidate variant genotype (*gt*) *sex* can predict MMVD graded disease status (*graded_status*: 0=healthy, 1=mild symptoms, 2=moderate symptoms, 3=severe symptoms). *P-values and* Beta coefficients (*β*) are presented for each individual explanatory variable, as well as combinations of explanatory variables. Significant p-values are written in bold and marked by an ‘*’.

**Table 21.** **MMVD association analysis using old controls and young cases in combined Swedish and Danish dachshund population.** Allele frequencies in the combined Swedish and Danish dachshund population at the nine candidate variants found to be segregating in dachshunds displayed in *i)* the entire dachshund population and subsamples of the data that include *ii)* controls aged >8 years and cases aged <10 years at screening, respectively; *iii):* controls aged >9 years and cases aged <9 years at screening, respectively; *iv):* controls aged >10 years and cases aged <8 years at screening, respectively; *v):* controls aged >11 years and cases aged <7 years at screening, respectively. Number of samples included (*n*) in each category (*Normal* and affected by *MMVD*, respectively) is stated and the p-value of a univariate linear regression analysis testing if genotype can predict disease status (*status*: 0=healthy, 1=case) in the dachshund populations. P-values <0.05 are marked with ‘*’. P-values <0.01 are written in bold and marked with ‘*’.

**Table 22.** **MMVD association analysis using old controls and young cases in Swedish dachshund population.** Allele frequencies in the Swedish dachshund population at the nine candidate variants found to be segregating in dachshunds displayed in *i)* the entire dachshund population and subsamples of the data that include *ii)* controls aged >8 years and cases aged <10 years at screening, respectively; *iii):* controls aged >9 years and cases aged <9 years at screening, respectively; *iv):* controls aged >10 years and cases aged <8 years at screening, respectively; *v):* controls aged >11 years and cases aged <7 years at screening, respectively. Number of samples included (*n*) in each category (*Normal* and affected by *MMVD*, respectively) is stated and the p-value of a univariate linear regression analysis testing if genotype can predict disease status (*status*: 0=healthy, 1=case) in the dachshund populations. P-values <0.05 are marked with ‘*’. P-values <0.01 are written in bold and marked with ‘*’.

**Table 23. Candidate gene heart expression raw data.** Quantification of mRNA levels of NEBL isoform nebulette, NEBL isoform LIM-nebulette and the neighboring *Mllt10* gene in tissue samples from mitral valve (n=23), papillary muscle (n=23) and left ventricular heart wall (n=21), from a total of 23 dogs representing 8 different breeds. Delta Delta C(T) values for the *Mllt10* assay (MLLT10_PAP2), two nebulette assays (Nebulette_4_PAP2, Nebulette_3_PAP2), two LIM-nebulette assays (LIM_N2_PAP2, LIM_N3_PAP2) and two reference gene assays, *GP3* (LIM_N2_GAP3_PAP) and *RPL13A* (LIM_N2_RPL13A_PAP), are presented as are the standard deviations for the target genes based on three technical replicates (SD_MLLT10_PAP2, SD_Nebulette_4_PAP2, SD_Nebulette_3_PAP2, SD_LIM_N2_PAP2, SD_LIM_N3_PAP2). *Breed*, age at sampling (*age)*, *healthy status* (where *normal* refers to a healthy individual, *MMVD* refers to an individual exhibiting MMVD symptoms, *MMVD chf* refers to an individual exhibiting MMVD symptoms and congestive heart failure and *DCM* refers to individuals affected by dilated cardiomyopathy) and *MMVD status* (0=healthy, 1 affected) and graded MMVD status, according to ACVIM criteria (0=A, 1=B1, 2=B2 symptoms, 3=C) is presented for each individual. RNA integrity values (RIN) are presented for each extraction and tissue. The last columns show *NEBL* candidate variant genotype in each individual.

**Table 24. Summary of heart eQTL analyses.** Results of linear regression analyses testing for associations between candidate *NEBL* variants (*Candidate variants*) and candidate gene (*Gene*) or candidate gene isoform (*isoform*) expression in three different heart tissues (*Mitral valve, Left ventricular heart wall and Papillary muscle*) are depicted. Analyses using two different primer pairs (*Primers*) were used for nebulette and LIM-nebulette, respectively and a single primer pair was used for *Mllt10*. Results of regression analyses are presented as p-values (.=p<0.1; *=p<0.05; **=p<0.01) for six different subsamples of the data characterized by employing varying stringencies for data inclusion as defined by RNA integrity numbers (*RIN-values*), technical replicate standard deviations (*sd*) and by including or excluding dogs affected by dilated cardiomyopathy (DCM). Sample size of each subsample (*n*) is presented. eQTLs that are consistent across all subsamples and primer pairs are highlighted in green.

**Table 25. TRAP and ENCODE regulatory element prediction. A)** *Raw* and *corrected p-values* for in-silico predictions of transcription factor binding to DNA sequence including 20 bp up- and downstream of candidate variants *NEBL* 1 and 4. *Matrix id* and *matrix name* of positive hits are presented. TRAP predicted no binding for *NEBL* 2 and 3. **B)** Results of SCREEN search for ENCODE Candidate cis-Regulatory Elements (cCREs)for the homologous human genome (hg38) position of NEBL 1, 2 , 3 and 4. *Start* and *end position*, *accession* and *type* of cCREs identified are presented. No cCREs were predicted for *NEBL* 2 and 3.

**Table 26. Age, sex and candidate allele frequencies in the general Swedish beagle population and the SLU beagle case control population.** *Population* (where *beagle Swedish* refers to the general Swedish beagle population, *beagle SLU all* refers to the complete beagle population at SLU, *beagle SLU MMVD* refers to individuals affected by MMVD at the SLU beagle population and *beagle SLU Normal* refers to healthy controls at the SLU beagle population); average age at screening (*Age*), sample size (*n*) and reference allele frequency at each of 10 candidate variants (stated beneath each candidate variant id, i.e. *NEBL 1, NEBL 2, NEBL 3, NEBL 4, NEBL 5, NEBL 6, LPHN2, HDGFL1, HTR1F*) are depicted. P-values are presented for univariate linear regressions investigating if candidate variant genotype (*GT*) can predict MMVD disease status (*status*: 0=healthy, 1=case). Significant associations are highlighted in yellow.

**Table 27. Regions under putative selection during cKCs breed formation.** Position (*start* and *end*) of the top 5% regions showing evidence of selection in cKCs as identified by *Sweepfinder 2*. *Scaffold* indicates chromosome for each selection region.

**Table 28. Primers and probes for TaqMan assays.** Annealing temperatures for amplification of each NEBL variant using PCR are indicated**.**

**Table 29. QPCR primers.**

**Table 30. EMSA oligonucleotides.**

**Table 31. *NEBL* 1 luciferase fragment primers.**

**Fig S1.**

**Fig S2.**

**Fig S3.**

**Fig S4.**

**Fig S5.**

**Fig S6.**

**Fig S7.**

**Fig S8.**

**Fig S9.**

**Fig S10.**

**Table 1.**

| **SNPs** | All | 13 737 824 |
| --- | --- | --- |
|  | Filtered | 11 899 463 |
|  | SNV detection power (%) | 97.82 |
|  | FDR (%) | 1.49 |
|  |  |  |
| **INDELs** | All | 4 213 281 |
|  | Filtered | 2 933 658 |
|  |  |  |
| **Genotypes** | GT accuracy (%) | 95.45 |
|  | Missingness (%) (for sites actually called) | 1.56 |
|  | Total missingness (%) (incl. false negative sites) | 2.97 |

**Table 2.**

|  | **Beagle** | **cKCs** | **German s.** | **golden r.** | **Labrador r.** | **s. poodle** | **Rottweiler** | **WHwt** |
| --- | --- | --- | --- | --- | --- | --- | --- | --- |
| **Heterozygosity** | 0,208 | 0,152 | 0,174 | 0,188 | 0,201 | 0,194 | 0,167 | 0,158 |
| **Total avg. pairwise FST** | 0,119 | 0,151 | 0,139 | 0,127 | 0,120 | 0,126 | 0,141 | 0,146 |

**Table 3.**

**Table 4.**

**Table 5.**

**Table 6.**

**Table 7.**

**Table 8.**

**Table 9.**

**Table 10.**

**Table 11.**

**Table 12.**

**Table 13.**

**Table 14.**

| **Avg. pairwise FST (VST for CNVs)** | **Variant type** | **beagle** | **cKCs** | **German s.** | **golden r.** | **Labrador r.** | **s. poodle** | **Rottweiler** | **WHwt** |
| --- | --- | --- | --- | --- | --- | --- | --- | --- | --- |
| 1 | All SNVs and INDELs | 4 | 71 | 3 | 1 | 0 | 0 | 91 | 9 |
| 1 | Derived, SNPs and INDELs at conserved sites | 2 | 2 | 0 | 0 | 0 | 0 | 0 | 0 |
|  |  |  |  |  |  |  |  |  |  |
| >=0.7 | All SNVs and INDELs | 3 154 | 111 165 | 54 622 | 14 118 | 4 028 | 7 385 | 65 405 | 93 321 |
| >=0.7 | Derived, SNPs and INDELs at conserved sites | 54 | 1 158 | 624 | 169 | 43 | 86 | 752 | 1 008 |
|  |  |  |  |  |  |  |  |  |  |
| >=0.7 | All large deletions | 1 | 35 | 17 | 3 | 0 | 4 | 20 | 25 |
| >=0.7 | Conserved deletions (phastcon>0) | 1 | 15 | 6 | 1 | 0 | 2 | 12 | 9 |
|  |  |  |  |  |  |  |  |  |  |
| >=0.7 | All CNVs | 0 | 4 | 0 | 1 | 1 | 0 | 1 | 2 |
| >=0.7 | Conserved CNVs (phastcon>0) | 0 | 2 | 0 | 1 | 1 | 0 | 1 | 2 |

**Table 15.**

| **Chr** | **Position** | **Phylop-score** | **avg. pairwise F_ST_** | **avg. pairwise F_ST_ rank** | **Breed with high F_ST_** | **total F_ST_/V_ST_** | **total F_ST_/V_ST_ rank** | **Status** | **Type** | **Gene** | **Phenotype** | **Reference** |
| --- | --- | --- | --- | --- | --- | --- | --- | --- | --- | --- | --- | --- |
| 4 | 67 040 939 | 7,3 | 1 | 1st | CKCS | 0.73 | 3rd | potentially causative | amino acid substitution | *GHR* | Size | *Genome Res.* 2013; 23:1985-95 |
| 7 | 43 787 274-43 804 023 | NA | - | - | - | 0.56 | 100th | potentially causative | deletion downstream of *SMAD2* | *SMAD2* | Size | *Genome Res.* 2013; 23:1985-95 |
| 18 | 48 413 694-48 415 206 | NA | - | - | - | 0.91 | 4th | causal | *FGF4* retrogene insertion | *FGF4* | Chondrodystrophy | *PNAS*. 2017 Oct; 114(43):11476-81 |
| 32 | 4 509 367 | 8,9 | - | - | - | 0.6 | 590th | likely causative | amino acid substitution | *FGF5* | Long, fluffy coat | *Anim Genet. 2006 Aug; 37(4):309-15.* |
| 27 | 2 539 211 | 4,1 | 0.96 | 1st | Poodle | 0.67 | 98th | likely causative | amino acid substitution | *KRT71* | Curly hair | *Science*. 2009 Oct; 326 (5949):150-3 |

**Table 16.**

|  |  |  |  |  |  |  |  |  |  | ***PhylopP*** | | **Average pairwise FST** | | | | | | | | **Reference allele frequency** | | | | | | | |
| --- | --- | --- | --- | --- | --- | --- | --- | --- | --- | --- | --- | --- | --- | --- | --- | --- | --- | --- | --- | --- | --- | --- | --- | --- | --- | --- | --- |
| **Variant id** | **Chr** | **Pos** | **Ref** | **Alt** | **Ancestral** | **Wolf alt AF** | **Gene** | **Expr. Fold change** | **SNPeff** | **100 vert.** | **46 mamm.** | **Beagle** | **cKCs** | **German s.** | **golden r.** | **Labrador r.** | **s. poodle** | **Rottweiler** | **WHwt** | **beagle** | **cKCs** | **German s.** | **golden r.** | **Labrador r.** | **s. poodle** | **Rottweiler** | **WHwt** |
| SORBS2 | 16 | 45 026 823 | C | T | C | 0 | *SORBS2* | -1.99 | INTRON/ NON_SYN | 10,0 | 2,8 | 0,12 | 0,82 | 0,12 | 0,12 | 0,12 | 0,12 | 0,12 | 0,12 | 1,00 | 0,18 | 1,00 | 1,00 | 1,00 | 1,00 | 1,00 | 1,00 |
| NEBL 3 | 2 | 11 979 724 | G | A | G | 0 | *NEBL* | -2.06 | INTRON | 2,8 | 2,5 | 0,39 | 0,75 | 0,23 | 0,29 | 0,25 | 0,25 | 0,39 | 0,27 | 1,00 | 0,00 | 0,80 | 0,46 | 0,55 | 0,57 | 1,00 | 0,89 |
| NEBL 4 | 2 | 12 082 890 | T | C | C | 1.00 | *NEBL* | -2.06 | INTRON | 2,8 | 1,5 | 0,14 | 0,77 | 0,19 | 0,21 | 0,14 | 0,15 | 0,15 | 0,22 | 0,18 | 0,98 | 0,08 | 0,41 | 0,24 | 0,28 | 0,14 | 0,05 |
| NEBL 5 | 2 | 12 165 498 | A | T | T | 1.00 | *NEBL* | -2.06 | INTRON | 2,3 | 1,0 | 0,18 | 0,80 | 0,29 | 0,26 | 0,19 | 0,21 | 0,20 | 0,26 | 0,13 | 0,95 | 0,41 | 0,00 | 0,08 | 0,26 | 0,05 | 0,00 |
| *NA* | 26 | 18 123 310 | C | T | C | 0 | *SSH1* | -1.85 | INTRON | 2,2 | 1,0 | 0,11 | 0,70 | 0,12 | 0,11 | 0,14 | 0,11 | 0,15 | 0,15 | 0,93 | 0,22 | 0,97 | 0,94 | 0,86 | 0,94 | 1,00 | 1,00 |

**Table 17.**

|  |  |  |  |  |  |  |  |  | ***PhyloP*** | | **Average pairwise F_ST_** | | | | | | | | **Reference allele frequency** | | | | | | | |
| --- | --- | --- | --- | --- | --- | --- | --- | --- | --- | --- | --- | --- | --- | --- | --- | --- | --- | --- | --- | --- | --- | --- | --- | --- | --- | --- |
| **Variant id** | **Chr** | **Pos** | **Ref** | **Alt** | **Ancestral** | **Wolf alt AF** | ***SNPeff*** | | **100 vert.** | **46 mamm.** | **Beagle** | **CKCS** | **German s.** | **Golden r.** | **Labrador r.** | **s. poodle** | **Rottweiler** | **WHwt** | **beagle** | **CKCS** | **German s.** | **Golden r.** | **Labrador r.** | **s. poodle** | **Rottweiler** | **WHwt** |
| NEBL 1 | 2 | 11 816 535 | A | G | A | 0,00 | INTERGENIC | | 4,1 | 2,3 | 0,26 | 0,72 | 0,17 | 0,19 | 0,17 | 0,22 | 0,19 | 0,36 | 0,90 | 0,00 | 0,64 | 0,80 | 0,68 | 0,48 | 0,55 | 0,97 |
| NEBL 2 | 2 | 11 823 576 | C | T | C | 0,33 | INTERGENIC | | 2,2 | 2,6 | 0,24 | 0,70 | 0,16 | 0,17 | 0,16 | 0,17 | 0,26 | 0,38 | 0,86 | 0,00 | 0,70 | 0,74 | 0,63 | 0,56 | 0,41 | 0,97 |
| NEBL 6 | 2 | 12 567 546 | A | G | A | 0,17 | INTERGENIC | | 0,5 | 2,0 | 0,16 | 0,90 | 0,17 | 0,22 | 0,20 | 0,16 | 0,16 | 0,20 | 0,95 | 0,03 | 0,97 | 0,78 | 1,00 | 0,92 | 0,89 | 1,00 |

**Table 18.**

|  |  |  | **chr** | 2 | 2 | 2 | 2 | 2 | 2 | 16 | 6 | 7 | 31 |  |  |  |
| --- | --- | --- | --- | --- | --- | --- | --- | --- | --- | --- | --- | --- | --- | --- | --- | --- |
|  |  |  | **pos** | 11 816 535 | 11 823 576 | 11 979 724 | 12 082 890 | 12 165 498 | 12 567 546 | 45 026 823 | 65 609 405 | 41 245 057 | 273 549 |  |  | **Heart disease incidence** |
|  |  | **Breed** | **SNV id** | **NEBL 1** | **NEBL 2** | **NEBL 3** | **NEBL 4** | **NEBL 5** | **NEBL 6** | **SORBS2** | **LPHN2** | **HDGFL1** | **HTR1F** | **Weight** | **MMVD incidence** |  |
|  |  |  | **n/ref.** | **A** | **C** | **G** | **T** | **A** | **A** | **C** | **T** | **A** | **T** |  |  |  |
| **Allele frequencies** | | beagle | 23 | 0,91 | 0,88 | 1 | 0,16 | 0,11 | 0,95 | 1 | 0,9 | 0,46 | 0,78 | 10.9 | 36.7 | 58.9 |
|  |  | cKCs | 35 | 0 | 0 | 0 | 0,99 | 0,94 | 0,03 | 0,25 | 0,17 | 0 | 0,15 | 7.0 | 333 | 433 |
|  |  | cocker spaniel | 25 | 0,14 | 0,45 | 0,64 | 0,38 | 0,23 | 0,73 | 1 | 1 | 0,08 | 1 | 13.6 | 24.9 | 57.6 |
|  |  | dachshund | 115 | 0,2 | 0,39 | 0,68 | 0,36 | 0,24 | 0,62 | 1 | 0,97 | 0,13 | 0,97 | 8.6 | 89.4 | 128 |
|  |  | Dobermann p. | 9 | 0,38 | 0,44 | 0,44 | 0,44 | 0,5 | 0,67 | 1 | 0,8 | 0,8 | 1 | 34.9 | 11.6 | 86.3 |
|  |  | German s. | 20 | 0,64 | 0,7 | 0,8 | 0,08 | 0,41 | 0,97 | 1 | 1 | 1 | 1 | 38.6 | 1.44 | 8.31 |
|  |  | golden r. | 20 | 0,8 | 0,74 | 0,46 | 0,41 | 0 | 0,78 | 1 | 1 | 0,88 | 1 | 29.5 | 4.77 | 17.1 |
|  |  | Grand Danois | 10 | 0,6 | 0,6 | 0,85 | 0,6 | 0,4 | 1 | 1 | 1 | 0,85 | 1 | 61.7 | 18.6 | 67.6 |
|  |  | Labrador r. | 20 | 0,68 | 0,63 | 0,55 | 0,24 | 0,08 | 1 | 1 | 0,97 | 0,74 | 1 | 30.6 | 2.27 | 9.49 |
|  |  | Norfolk t. | 11 | 0,36 | 0,44 | 1 | 0,05 | 0,15 | 0,69 | 1 | 1 | 0,79 | 1 | 5.4 | 217 | 319 |
|  |  | Norwich t. | 11 | 0,55 | 0,59 | 1 | 0,55 | 0,59 | 0,85 | 1 | 1 | 0,55 | 0,9 | 5.4 | 7.66 | 38.3 |
|  |  | s. poodle | 20 | 0,48 | 0,56 | 0,57 | 0,28 | 0,26 | 0,92 | 1 | 0,95 | 0,39 | 1 | 26.1 | 19.4 | 34.2 |
|  |  | Rottweiler | 20 | 0,55 | 0,41 | 1 | 0,14 | 0,05 | 0,89 | 1 | 1 | 0,61 | 1 | 45.4 | 3.84 | 12.0 |
|  |  | WHwt | 20 | 0,97 | 0,97 | 0,89 | 0,05 | 0 | 1 | 1 | 1 | 0,95 | 0,95 | 8.4 | 8.35 | 27.9 |
| **Regression models** |  | lm(MMVD incidence~weight) | **p-value** |  |  |  |  |  |  |  |  |  |  | (0,092) |  |  |
|  |  |  | **𝜷** |  |  |  |  |  |  |  |  |  |  | -2,615 |  |  |
|  |  |  | **intercept** |  |  |  |  |  |  |  |  |  |  | 116,554 |  |  |
|  |  |  | **Adj. R2** |  |  |  |  |  |  |  |  |  |  | 0,15 |  |  |
|  |  |  |  |  |  |  |  |  |  |  |  |  |  |  |  |  |
|  | **Incl. cKCs** | lm(MMVD incidence~gt) | **p-value** | **0,018** | **0,005** | (0,112) | (0,093) | **0,05** | **0,0001** | **0,0002** | **0,0004** | (0,086) | **0,0004** |  |  |  |
|  |  |  | **𝜷** | -215,72 | -289,31 | -150,7 | 176,838 | 197,777 | -326,8 | -398,26 | -342,78 | -140,46 | -335,13 |  |  |  |
|  |  |  | **intercept** | 167,5 | 216,82 | 161,99 | -4,108 | -0,3048 | 314,7 | 432,57 | 368,06 | 138,21 | 360,85 |  |  |  |
|  |  |  | **Adj. R2** | 0,34 | 0,45 | 0,13 | 0,15 | 0,22 | 0,7 | 0,63 | 0,56 | 0,16 | 0,57 |  |  |  |
|  |  |  |  |  |  |  |  |  |  |  |  |  |  |  |  |  |
|  |  | lm(Heart disease incidence~gt) | **p-value** | **0,001** | **0,0005** | **0,023** | (0,08) | **0,037** | **0,0001** | **0,0003** | **0,0005** | **0,014** | **0,0007** |  |  |  |
|  |  |  | **𝜷** | -287,89 | -378,23 | -187,91 | 235,76 | 268,52 | -420,35 | -488,6 | -431,7 | -165,48 | -407,6 |  |  |  |
|  |  |  | **intercept** | 241,99 | 303,42 | 225,3 | 13,04 | 16,74 | 425,97 | 555,2 | 486,2 | 189,97 | 463,9 |  |  |  |
|  |  |  | **Adj. R2** | 0,37 | 0,46 | 0,12 | 0,17 | 0,26 | 0,7 | 0,57 | 0,53 | 0,12 | 0,5 |  |  |  |
|  |  |  |  |  |  |  |  |  |  |  |  |  |  |  |  |  |
|  | **Excl. cKCs** | lm(MMVD incidence~gt) | **p-value** | (0,18) | (0,24) | (0,31) | (0,31) | (0,71) | (0,067) | NA | (0,74) | (0,69) | (0,90) |  |  |  |
|  |  |  | **𝜷** | -93,39 | -115,65 | 85,43 | -97,49 | -35,98 | -225,93 | NA | 304,97 | -24,4 | 35,99 |  |  |  |
|  |  |  | **intercept** | 86,46 | 103,69 | -30,62 | 62,35 | 42,66 | 226,69 | 34,3 | -67,62 | 49,75 | -0,584 |  |  |  |
|  |  |  | **Adj. R2** | 0,08 | 0,05 | -0,08 | 0,01 | -0,08 | 0,21 | NA | -0,11 | -0,08 | -0,09 |  |  |  |
|  |  |  |  |  |  |  |  |  |  |  |  |  |  |  |  |  |
|  |  | lm(Heart disease incidence~gt) | **p-value** | (0,13) | (0,17) | (0,40) | (0,56) | (0,95) | **0,038** | NA | (0,92) | (0,81) | (0,82) |  |  |  |
|  |  |  | **𝜷** | -146,71 | -184,54 | 98,88 | -80,09 | 8,66 | -349,8 | NA | -41,42 | -20,67 | 92,6 |  |  |  |
|  |  |  | **intercept** | 148,45 | 177,24 | -8,63 | 89,56 | 64,5 | 362.3 | 66,52 | 106,63 | 79,6 | -23,24 |  |  |  |
|  |  |  | **Adj. R2** | 0,12 | 0,09 | -0,02 | -0,06 | -0,09 | 0,27 | NA | -0,09 | -0,08 | -0,09 |  |  |  |
|  |  |  |  |  |  |  |  |  |  |  |  |  |  |  |  |  |
|  |  |  | **P-value (model)** | (0,14) | (0,1) | (0,24) | (0,26) | (0,32) | (0,12) | (0,13) | (0,31) | (0,32) | (0,20) |  |  |  |
|  |  | lm(MMVD incidence~gt+weight) | **p-value (gt)** | (0,21) | (0,13) | (0,45) | (0,50) | (0,85) | (0,17) | NA | (0,77) | (0,81) | (0,34) |  |  |  |
|  |  |  | **𝜷(gt)** | -83,6 | -136,5 | 62,3 | -63,9 | -16,9 | -178 | NA | 87,6 | 15 | 284,9 |  |  |  |
|  |  |  | **𝜷(weight)** | -1,39 | -1,7 | -1,4 | -1,3 | -1,5 | -0,98 | -1,5 | -1,6 | -1,6 | -1,99 |  |  |  |
|  |  |  | **intercept** | 115,2 | 157,8 | 20,3 | 85,5 | 74,7 | 210 | 71,4 | -13,6 | 64,5 | -193,1 |  |  |  |
|  |  |  | **Adj. R2** | 0,19 | 0,25 | 0,1 | 0,08 | 0.04 | 0,21 | 0,13 | 0,05 | 0,05 | 0.13 |  |  |  |

**Table 19.**

|  | **n** | **age** | **sex (f/m)** | **NEBL1** | **NEBL2** | **NEBL3** | **NEBL4** | **NEBL5** | **NEBL6** | **LPHN2** | **HDGFL1** | **HTR1F** |
| --- | --- | --- | --- | --- | --- | --- | --- | --- | --- | --- | --- | --- |
| **Controls** | 43 | 8,8 | 21/22 | 0,18 | 0,44 | 0,67 | 0,35 | 0,23 | 0,61 | 0,94 | 0,12 | 0,97 |
| **MMVD** | 79 | 10,7 | 36/43 | 0,21 | 0,36 | 0,70 | 0,37 | 0,23 | 0,61 | 0,99 | 0,14 | 0,97 |
| lm(status~x) p-value | | 3,80E-05 | ns | ns | ns | ns | ns | ns | ns | 0,044* | ns | ns |
| β |  | 0,071 |  |  |  |  |  |  |  | -0,378 |  |  |
| R^2^ |  | 0,125 |  |  |  |  |  |  |  | 0,027 |  |  |
| lm(graded_status~x) p-value | | 6,40E-06 | ns | ns | ns | ns | ns | ns | ns | ns | ns | ns |
| β |  | 0,133 |  |  |  |  |  |  |  |  |  |  |
| R^2^ |  | 0,15 |  |  |  |  |  |  |  |  |  |  |

**Table 20.**

| *i)* | **lm(graded_status=gt*age)** | | |  | *ii)* | **lm(graded_status=gt*age*sex)** | | | |  |  |  |
| --- | --- | --- | --- | --- | --- | --- | --- | --- | --- | --- | --- | --- |
| ***NEBL1*** | intercept=-0.351 | |  |  | ***NEBL1*** | Intercept=0.548 | |  |  |  |  |  |
|  | adjR2=0.120 | |  |  |  | adjR2=0.094 | |  |  |  |  |  |
|  | gt | age | gt*age |  |  | gt | age | sex | gt*age | gt*sex | age*sex | gt*age*sex |
| 𝛽 | 0.003 | 0.134 | -0.007 |  | 𝛽 | -0.569 | 0.058 | -1.332 | 0.041 | 0.900 | 0.114 | -0.077 |
| p-value | ns | ns | ns |  | p-value | ns | ns | ns | ns | ns | ns | ns |
|  |  |  |  |  |  |  |  |  |  |  |  |  |
|  |  |  |  |  |  |  |  |  |  |  |  |  |
| ***NEBL2*** | intercept=-0.203 | |  |  | ***NEBL2*** | intercept=0.720 | |  |  |  |  |  |
|  | adjR2=0.144 | |  |  |  | adjR2=0.157 | |  |  |  |  |  |
|  | gt | age | gt*age |  |  | gt | age | sex | gt*age | gt*sex | age*sex | gt*age*sex |
| 𝛽 | -0.197 | 0.096 | -0.031 |  | 𝛽 | -1.016 | -0.005 | -1.967 | 0.119 | 1.681 | 0.217 | -0.182 |
| p-value | ns | ns | ns |  | p-value | 0.098 | ns | ns | **0.043*** | 0.051 | 0.071 | **0.029*** |
|  |  |  |  |  |  |  |  |  |  |  |  |  |
|  |  |  |  |  |  |  |  |  |  |  |  |  |
| ***NEBL3*** | intercept=0.202 | |  |  | ***NEBL3*** | intercept=0.502 | |  |  |  |  |  |
|  | adjR2=0.159 | |  |  |  | adjR2=0.142 | |  |  |  |  |  |
|  | gt | age | gt*age |  |  | gt | age | sex | gt*age | gt*sex | age*sex | gt*age*sex |
| 𝛽 | -0.944 | 0.066 | 0.104 |  | 𝛽 | -1.260 | 0.024 | -0.446 | 0.147 | 0.532 | 0.064 | -0.073 |
| p-value | **0.043*** | ns | **0.027*** |  | p-value | 0.066 | ns | ns | **0.034*** | ns | ns | ns |
|  |  |  |  |  |  |  |  |  |  |  |  |  |
|  |  |  |  |  |  |  |  |  |  |  |  |  |
| ***NEBL4*** | intercept=0.647 | |  |  | ***NEBL4*** | intercept=1.908 | |  |  |  |  |  |
|  | adjR2=0.179 | |  |  |  | adjR2=0.191 | |  |  |  |  |  |
|  | gt | age | gt*age |  |  | gt | age | sex | gt*age | gt*sex | age*sex | gt*age*sex |
| 𝛽 | -0.914 | 0.022 | 0.096 |  | 𝛽 | -1.836 | -0.113 | -2.358 | 0.195 | 1.767 | 0.254 | -0.190 |
| p-value | 0.052 | ns | **0.033*** |  | p-value | **0.009*** | ns | ns | **0.003*** | 0.062 | 0.051 | **0.034*** |
|  |  |  |  |  |  |  |  |  |  |  |  |  |
|  |  |  |  |  |  |  |  |  |  |  |  |  |
| ***NEBL5*** | intercept=-0.574 | |  |  | ***NEBL5*** | intercept=-1.270 | |  |  |  |  |  |
|  | adjR2=0.136 | |  |  |  | adjR2=0.133 | |  |  |  |  |  |
|  | gt | age | gt*age |  |  | gt | age | sex | gt*age | gt*sex | age*sex | gt*age*sex |
| 𝛽 | 0.075 | 0.150 | -0.010 |  | 𝛽 | 0.520 | 0.235 | 3.144 | -0.066 | -1.754 | -0.331 | 0.191 |
| p-value | ns | 0.091 | ns |  | p-value | ns | **0.022*** | ns | ns | ns | ns | ns |
|  |  |  |  |  |  |  |  |  |  |  |  |  |
|  |  |  |  |  |  |  |  |  |  |  |  |  |
| ***NEBL6*** | intercept=-0.987 | |  |  | ***NEBL6*** | intercept=-1.274 | |  |  |  |  |  |
|  | adjR2=0.158 | |  |  |  | adjR2=0.145 | |  |  |  |  |  |
|  | gt | age | gt*age |  |  | gt | age | sex | gt*age | gt*sex | age*sex | gt*age*sex |
| 𝛽 | 0.741 | 0.196 | -0.081 |  | 𝛽 | 1.071 | 0.230 | 0.768 | -0.124 | -0.941 | -0.092 | 0.116 |
| p-value | ns | **3.97e-5** | 0.090 |  | p-value | ns | **0.0004*** | ns | 0.063 | ns | ns | ns |
|  |  |  |  |  |  |  |  |  |  |  |  |  |
|  |  |  |  |  |  |  |  |  |  |  |  |  |
| ***LPHN2*** | intercept=-0.331 | |  |  | ***LPHN2*** | intercept=-0.345 | |  |  |  |  |  |
|  | adjR2=0.144 | |  |  |  | adjR2=0.115 | |  |  |  |  |  |
|  | gt | age | gt*age |  |  | gt | age | sex | gt*age | gt*sex | age*sex | gt*age*sex |
| 𝛽 | -0.813 | 0.124 | 0.036 |  | 𝛽 | -1.241 | 0.124 | 0.016 | 0.087 | 0.781 | 0.0005 | -0.093 |
| p-value | ns | **8.2e-5*** | ns |  | p-value | ns | **0.007*** | ns | ns | ns | ns | ns |
|  |  |  |  |  |  |  |  |  |  |  |  |  |
|  |  |  |  |  |  |  |  |  |  |  |  |  |
| ***HDGFL1*** | intercept=-0.822 | |  |  | ***HDGFL1*** | intercept=1.063 | |  |  |  |  |  |
|  | adjR2=0.1361 | |  |  |  | adjR2=0.124 | |  |  |  |  |  |
|  | gt | age | gt*age |  |  | gt | age | sex | gt*age | gt*sex | age*sex | gt*age*sex |
| 𝛽 | -0.694 | 0.003 | -0.074 |  | 𝛽 | -0.888 | -0.055 | -0.531 | 0.109 | 0.445 | 0.118 | -0.080 |
| p-value | ns | ns | ns |  | p-value | ns | ns | ns | ns | ns | ns | ns |
|  |  |  |  |  |  |  |  |  |  |  |  |  |
|  |  |  |  |  |  |  |  |  |  |  |  |  |
| ***HTR1F*** | intercept=-0.441 | |  |  | ***HTR1F*** | intercept=-0.445 | |  |  |  |  |  |
|  | adjR2=0.140 | |  |  |  | adjR2=0.129 | |  |  |  |  |  |
|  | gt | age | gt*age |  |  | gt | age | sex | gt*age | gt*sex | age*sex | gt*age*sex |
| 𝛽 | 0.124 | 0.134 | -0.033 |  | 𝛽 | -0.317 | 0.132 | 0.023 | 0.018 | 8.266 | 0.004 | -0.905 |
| p-value | ns | **1.02e-5*** | ns |  | p-value | ns | **0.004*** | ns | ns | ns | ns | ns |

**Table 21.**

| **All dachshunds (SWE+DK)** | **age** | **n** | **NEBL1** | **NEBL2** | **NEBL3** | **NEBL4** | **NEBL5** | **NEBL6** | **LPHN2** | **HDGFL1** | **HTR1F** |
| --- | --- | --- | --- | --- | --- | --- | --- | --- | --- | --- | --- |
| Normal | 10,3 | 52 | 0,16 | 0,40 | 0,66 | 0,34 | 0,23 | 0,63 | 0,94 | 0,12 | 0,95 |
| MMVD | 9,2 | 99 | 0,19 | 0,36 | 0,71 | 0,39 | 0,24 | 0,59 | 0,98 | 0,15 | 0,95 |
| lm(status~GT) |  |  | n.s. | n.s. | n.s. | n.s. | n.s. | n.s. | 0,041* | n.s. | n.s. |
|  |  |  |  |  |  |  |  |  |  |  |  |
| **Old cases vs. young controls (SWE+DK)** | **age** | **n** | **NEBL11** | **NEBL7** | **NEBL2** | **NEBL4** | **NEBL8** | **NEBL5** | **LPHN2** | **HDGFL1** | **HTR1F** |
| Nomral | >8 | 34 | 0,21 | 0,46 | 0,72 | 0,43 | 0,26 | 0,59 | 0,95 | 0,14 | 0,94 |
| MMVD | <10 | 48 | 0,16 | 0,29 | 0,69 | 0,33 | 0,26 | 0,54 | 0,99 | 0,16 | 0,95 |
| lm(status~GT) |  |  | n.s. | 0,048* | n.s. | n.s. | n.s. | n.s. | n.s. | n.s. | n.s. |
|  |  |  |  |  |  |  |  |  |  |  |  |
| Nomral | >9 | 22 | 0,20 | 0,50 | 0,73 | 0,39 | 0,30 | 0,57 | 0,98 | 0,14 | 0,93 |
| MMVD | <9 | 28 | 0,14 | 0,25 | 0,68 | 0,30 | 0,21 | 0,61 | 0,98 | 0,18 | 0,93 |
| lm(status~GT) |  |  | n.s. | 0,019* | n.s. | n.s. | n.s. | n.s. | n.s. | n.s. | n.s. |
|  |  |  |  |  |  |  |  |  |  |  |  |
| Nomral | >10 | 14 | 0,25 | 0,57 | 0,79 | 0,50 | 0,36 | 0,50 | 0,96 | 0,14 | 0,93 |
| MMVD | <8 | 16 | 0,19 | 0,22 | 0,69 | 0,31 | 0,22 | 0,69 | 1,00 | 0,25 | 1,00 |
| lm(status~GT) |  |  | n.s. | 0,015* | n.s. | n.s. | n.s. | n.s. | n.s. | n.s. | n.s. |
|  |  |  |  |  |  |  |  |  |  |  |  |
| Nomral | >11 | 10 | 0,25 | 0,60 | 0,85 | 0,60 | 0,30 | 0,45 | 0,95 | 0,20 | 0,95 |
| MMVD | <7 | 8 | 0,06 | 0,13 | 0,75 | 0,31 | 0,13 | 0,69 | 1,00 | 0,50 | 1,00 |
| lm(status~GT) |  |  | n.s. | **0,008**** | n.s. | 0,095 | n.s. | n.s. | n.s. | n.s. | n.s. |

**Table 22.**

| **All dachshunds (SWE)** | **age** | **n** | **NEBL1** | **NEBL2** | **NEBL3** | **NEBL4** | **NEBL5** | **NEBL6** | **LPHN2** | **HDGFL1** | **HTR1F** |
| --- | --- | --- | --- | --- | --- | --- | --- | --- | --- | --- | --- |
| Normal |  | 43 | 0,18 | 0,44 | 0,69 | 0,35 | 0,24 | 0,61 | 0,94 | 0,12 | 0,96 |
| MMVD |  | 79 | 0,20 | 0,36 | 0,70 | 0,38 | 0,23 | 0,61 | 0,99 | 0,14 | 0,97 |
| lm(status~GT) |  |  | n.s. | n.s. | n.s. | n.s. | n.s. | n.s. | 0,04* | n.s. | n.s. |
|  |  |  |  |  |  |  |  |  |  |  |  |
|  |  |  |  |  |  |  |  |  |  |  |  |
|  |  |  |  |  |  |  |  |  |  |  |  |
| **Old cases vs. young controls (SWE)** | **age** | **n** | **NEBL1** | **NEBL2** | **NEBL3** | **NEBL4** | **NEBL5** | **NEBL6** | **LPHN2** | **HDGFL1** | **HTR1F** |
| Normal | >8 | 24 | 0,25 | 0,54 | 0,79 | 0,48 | 0,29 | 0,54 | 0,96 | 0,15 | 0,96 |
| MMVD | <10 | 32 | 0,17 | 0,28 | 0,64 | 0,30 | 0,27 | 0,56 | 1,00 | 0,14 | 0,97 |
| lm(status~GT) |  |  | n.s. | **0,007**** | 0,074 | 0,042* | n.s. | n.s. | 0,098 | n.s. | n.s. |
|  |  |  |  |  |  |  |  |  |  |  |  |
|  |  |  |  |  |  |  |  |  |  |  |  |
| Normal | >9 | 18 | 0,22 | 0,53 | 0,78 | 0,42 | 0,31 | 0,53 | 0,97 | 0,17 | 0,94 |
| MMVD | <9 | 18 | 0,19 | 0,25 | 0,61 | 0,25 | 0,19 | 0,68 | 1,00 | 0,14 | 0,94 |
| lm(status~GT) |  |  | n.s. | 0,026* | 0,095 | n.s. | n.s. | n.s. | n.s. | n.s. | n.s. |
|  |  |  |  |  |  |  |  |  |  |  |  |
|  |  |  |  |  |  |  |  |  |  |  |  |
| Normal | >10 | 12 | 0,25 | 0,58 | 0,79 | 0,54 | 0,38 | 0,46 | 0,96 | 0,17 | 0,96 |
| MMVD | <8 | 11 | 0,23 | 0,23 | 0,64 | 0,27 | 0,18 | 0,77 | 1,00 | 0,18 | 1,00 |
| lm(status~GT) |  |  | n.s. | 0,038* | n.s. | 0,071 | n.s. | 0,037* | n.s. | n.s. | n.s. |
|  |  |  |  |  |  |  |  |  |  |  |  |
| Normal | >11 | 8 | 0,25 | 0,625 | 0,875 | 0,6875 | 0,3125 | 0,375 | 0,9375 | 0,25 | 1 |
| MMVD | <7 | 4 | 0,125 | 0,125 | 0,75 | 0,25 | 0 | 0,875 | 1 | 0,5 | 1 |
| lm(status~GT) |  |  | n.s. | 0,031* | n.s. | 0,023* | n.s. | 0,031* | n.s. | n.s. | n.s. |

**Table 23.**

| **Sample Name** | **SD_MLLT10_PAP2** | **MLLT10_PAP2** | **SD_Nebulette_4_PAP2** | **Nebulette_4_PAP2** | **SD_Nebulette_3_PAP2** | **Nebulette_3_PAP2** | **LIM_N2_PAP2** | **SD_LIM_N2_PAP2** | **LIM_N3_PAP2** | **SD_LIM_N3_PAP2** | **LIM_N2_GAP3_PAP** | **LIM_N2_RPL13A_PAP** | **Dog_id** | **Breed** | **Age** | **Health status** | **MMVD status** | **graded MMVD status** | **Mitral_valve_RIN** | **Left_ventricular_heartwall_RIN** | **Papillary_muscle_RIN** | **NEBL1_A** | **NEBL2_C** | **NEBL3_G** | **NEBL4_T** | **NEBL5_A** | **NEBL6_A** |
| --- | --- | --- | --- | --- | --- | --- | --- | --- | --- | --- | --- | --- | --- | --- | --- | --- | --- | --- | --- | --- | --- | --- | --- | --- | --- | --- | --- |
| 40P | 0,186 | 0,647 | 0,126 | 1,003 | 0,280 | 1,123 | 0,975 | 0,019 | 1,120 | 0,147 | 1,346 | 0,826 | 1 | Jack Russel terrier | 9.5 | MMVD, chf | 1 | 2 | 7.6 | NA | 8.3 | 0 | 0 | 0 | 0 | 2 | 0 |
| 41P | 0,110 | 0,977 | 0,262 | 0,826 | 0,481 | 1,006 | 3,422 | 0,161 | 3,514 | 0,143 | 3,333 | 4,187 | 2 | Lapsk vallhund | 1.5 | Normal | 0 | 0 | 7.5 | 8.3 | 8.1 | 0 | 0 | 0 | 2 | 2 | 0 |
| 28P | 0,109 | 1,000 | 0,127 | 1,000 | 0,096 | 1,000 | 1,000 | 0,208 | 1,000 | 0,107 | 1,000 | 1,000 | 3 | beagle | 2 | Normal | 0 | 0 | 8.6 | 8 | 8 | 0 | 0 | 0 | 2 | 2 | 0 |
| 29P | 0,141 | 0,540 | 0,042 | 0,279 | 0,096 | 0,316 | 0,301 | 0,303 | 0,402 | 0,135 | 1,473 | 0,140 | 4 | beagle | 2 | Normal | 0 | 0 | 8.5 | 8 | 8 | 0 | 0 | 0 | 2 | 2 | 0 |
| 30P | 0,416 | 1,480 | 0,090 | 0,860 | 0,076 | 0,999 | 2,955 | 0,155 | 2,206 | 0,145 | 2,673 | 3,185 | 5 | beagle | 2 | Normal | 0 | 0 | 8.5 | 8 | 8 | 0 | 0 | 0 | 2 | 2 | 0 |
| 39P | 0,299 | 0,536 | 0,290 | 0,592 | 0,238 | 0,646 | 0,902 | 0,336 | 0,953 | 0,173 | 1,474 | 0,745 | 6 | cocker spaniel | 11.5 | MMVD | 1 | 1 | 7.7 | 8.8 | 8.8 | 2 | 2 | 2 | 0 | 0 | 2 |
| 31P | 0,147 | 0,535 | 0,063 | 0,248 | 0,086 | 0,290 | 0,880 | 0,126 | 0,531 | 0,726 | 1,524 | 0,693 | 7 | cKCs | 8.53 | MMVD | 1 | 1 | 8.4 | 8.4 | 9.1 | 2 | 2 | 2 | 0 | 0 | 2 |
| 32P | 0,309 | 0,636 | 0,205 | 0,704 | 0,034 | 0,856 | 0,837 | 0,103 | 0,765 | 0,341 | 1,399 | 0,622 | 8 | Dobermann p. | 4.9 | Normal | 0 | 0 | 9.1 | 8.4 | 8.5 | 1 | 0 | 1 | 1 | 1 | NA |
| 33P | 0,063 | 0,652 | 0,142 | 0,378 | 0,342 | 0,423 | 1,304 | 0,260 | 1,090 | 0,331 | 2,393 | 1,151 | 9 | cKCs | 8.26 | MMVD, chf | 1 | 2 | 4.4 | 7.9 | 6.6 | 2 | 2 | 2 | 0 | 0 | 2 |
| GD1_P | 0,116 | 1,001 | 0,069 | 0,338 | 0,087 | 0,653 | 1,209 | 0,242 | 1,518 | 0,242 | 0,830 | 1,546 | 10 | Grand Danois | 3.21 | Normal | 0 | NA | 7.2 | 7.9 | 7.9 | 0 | 1 | 1 | 2 | 2 | 0 |
| GD8_P | 0,362 | 1,637 | 0,063 | 0,506 | 0,019 | 1,149 | 2,503 | 0,228 | 2,608 | 0,201 | 1,884 | 2,569 | 11 | Grand Danois | 5.07 | Normal, DCM | 0 | NA | 6.4 | 8.7 | 5 | 2 | 2 | 0 | 2 | 2 | 0 |
| 34P_GD6_P | 0,249 | 0,603 | 0,066 | 0,537 | 0,223 | 0,586 | 1,157 | 0,094 | 1,617 | 0,133 | 0,777 | 0,622 | 12 | Grand Danois | 6.23 | Normal | 0 | 0 | 8.0 | 8.5 | 8.9 | 1 | 0 | 0 | 1 | 1 | 0 |
| 35P | 0,190 | 0,664 | 0,110 | 0,254 | 0,023 | 0,472 | 1,356 | 0,128 | 1,445 | 0,132 | 1,872 | 1,012 | 13 | cKCs | 15 | MMVD | 1 | 1 | 7.7 | 8.2 | 9.1 | 2 | 2 | 2 | 0 | 0 | 2 |
| 36P | 0,068 | 0,527 | 0,101 | 0,318 | 0,087 | 0,343 | 0,589 | 0,240 | 0,630 | 0,274 | 1,179 | 0,478 | 14 | cKCs | 2.86 | Normal | 0 | 0 | 6.5 | 5.5 | 5.8 | 2 | 2 | 2 | 0 | 0 | 2 |
| GD7_P | 0,221 | 1,463 | 0,184 | 0,244 | 0,285 | 0,533 | 2,281 | 0,066 | 1,023 | 0,630 | 1,594 | 2,114 | 15 | Grand Danois | 1.52 | Normal | 0 | NA | 8.2 | 6.1 | 6.1 | 2 | 0 | 0 | 1 | 2 | 0 |
| GD10_P | 0,307 | 1,609 | 0,132 | 0,303 | 0,607 | 0,560 | 1,404 | 0,421 | 1,436 | 0,459 | 1,207 | 1,777 | 16 | Grand Danois | 5.96 | Normal, DCM | 0 | NA | 7.1 | NA | 5.4 | 0 | 1 | 1 | 1 | 2 | 0 |
| GD5_P | 0,356 | 0,822 | 0,104 | 0,215 | 0,199 | 0,379 | 0,920 | 0,568 | 0,764 | 0,346 | 0,413 | 2,609 | 17 | Grand Danois | 11.09 | Normal | 0 | NA | 8.2 | 6.5 | 100 | 1 | 2 | 1 | 1 | 1 | 0 |
| GD4_P | 0,097 | 1,547 | 0,159 | 0,295 | 0,160 | 0,549 | 2,267 | 0,380 | 4,123 | 0,328 | 1,262 | 4,853 | 18 | Grand Danois | 8 | Normal, DCM | 0 | NA | 5.7 | 6.4 | 6.5 | 2 | 2 | 0 | 0 | 0 | 0 |
| GD9_P | 0,556 | 1,469 | 0,424 | 0,124 | 0,216 | 0,240 | NA | NA | NA | NA | NA | NA | 19 | Grand Danois | 0.83 | Normal | 0 | NA | 7.5 | 6.1 | 4.7 | 0 | 0 | 0 | 0 | 0 | 0 |
| GD2_P | 0,123 | 2,842 | 0,043 | 0,338 | 0,086 | 0,532 | 1,309 | 0,102 | 0,777 | 0,434 | 0,360 | 2,577 | 20 | Grand Danois | 2.25 | Normal, DCM | 0 | NA | 5.4 | 5.9 | 6 | 0 | 0 | 0 | 0 | 1 | 0 |
| GD3_P | 0,240 | 1,608 | 0,050 | 0,646 | 0,085 | 0,896 | 2,083 | 0,255 | 1,688 | 0,339 | 1,625 | 3,516 | 21 | Grand Danois | 1.59 | Normal | 0 | NA | 7.9 | 8.6 | 8.3 | 0 | 0 | 0 | 0 | 1 | 0 |
| 37P | 0,203 | 0,356 | 0,089 | 0,175 | 0,022 | 0,192 | 0,380 | 0,442 | 0,449 | 0,652 | 1,083 | 0,192 | 22 | cKCs | 8.7 | Normal | 0 | 0 | 7.6 | 8 | 8 | 2 | 2 | 2 | 0 | 0 | 2 |
| 38P | 0,173 | 0,775 | 0,225 | 0,461 | 0,142 | 0,762 | 1,601 | 0,240 | 1,253 | 0,554 | 3,036 | 1,274 | 23 | dachshund | 16 | MMVD, chf | 1 | 2 | 5.5 | 8 | 8 | 2 | 1 | 0 | 0 | 0 | 2 |

**Table 24.**

**Table 25.**

| **a)** | TRAP analysis for NEBL 1 reference ("A") |  |  |  |  |  |
| --- | --- | --- | --- | --- | --- | --- |
|  | **Raw P-value** | **Corrected p-value (Benjamini-Hochberg)** | **Matrix ID** | **Matrix name** |  |  |
|  | <0.0001 | 0.037 | M00100 | V$CDXA_01 |  |  |
|  | <0.0001 | 0.037 | M00489 | V$NKX62_Q2 |  |  |
|  | 0.0001 | 0.037 | M01420 | V$NCX_02 |  |  |
|  | 0.0002 | 0.04 | M01432 | V$HOXD8_01 |  |  |
|  | 0.0002 | 0.04 | M00423 | V$FOXJ2_02 |  |  |
|  |  |  |  |  |  |  |
|  |  |  |  |  |  |  |
|  | TRAP analysis for NEBL 4 reference ("T") |  |  |  |  |  |
|  | **Raw P-value** | **Corrected p-value (Benjamini-Hochberg)** | **Matrix ID** | **Matrix name** |  |  |
|  | <0.0002 | 0.04 | M00100 | V$CDXA_01 |  |  |
|  | <0.0002 | 0.04 | M00747 | V$IRF1_Q6 |  |  |
|  | <0.0002 | 0.04 | M01659 | V$CDX2_Q5_01 |  |  |
|  | 0.0002 | 0.04 | M00394 | V$MSX1_01 |  |  |
|  |  |  |  |  |  |  |
|  |  |  |  |  |  |  |
| **b)** | hg38 ENCODE Candidate cis-Regulatory Elements (cCRE) by ENCODE |  |  |  |  |  |
|  | **Variant** | **hg38 chr** | **start cCRE in hg 38** | **end cCRE in hg 38** | **cCRE accession** | **type** |
|  | *NEBL 1* | chr10 | 21270017 | 21270256 | EH38E1453677 | distal enhancer like signature |
|  | *NEBL 4* | chr10 | 20948574 | 20948913 | EH38E1453481 | distal enhancer like signature |
|  |  |  |  |  |  |  |

**Table 26.**

| **Population** | **Age** | **n** | **NEBL1** | **NEBL2** | **NEBL3** | **NEBL4** | **NEBL5** | **NEBL6** | **LPHN2** | **HDGFL1** | **HTR1F** |
| --- | --- | --- | --- | --- | --- | --- | --- | --- | --- | --- | --- |
| beagle Swedish | NA | 23 | 0,91 | 0,88 | 1 | 0,16 | 0,11 | 0,95 | 0,89 | 0,46 | 0,78 |
| beagle SLU all | 8,8 | 22 | 0,77 | 0,84 | 0,95 | 0,07 | 0,09 | 1 | 0,82 | 0,5 | 0,84 |
| beagle SLU MMVD | 10,8 | 10 | 0,6 | 0,75 | 0,89 | 0,1 | 0,15 | 1 | 0,8 | 0,35 | 0,9 |
| beagle SLU Normal | 7,1 | 12 | 0,92 | 0,92 | 1 | 0,04 | 0,04 | 1 | 0,83 | 0,63 | 0,79 |
| lm(status~GT) |  |  | 0,042* | 0,104 | 0,11 | n.s. | n.s. | n.s. | n.s. | 0,118 | n.s. |

**Table 27.**

| Scaffold | start | end |
| --- | --- | --- |
| chr_1 | 68323373 | 68693399 |
| chr_1 | 114967949 | 115367979 |
| chr_1 | 115768028 | 115938035 |
| chr_3 | 1500396 | 1730408 |
| chr_3 | 9491152 | 12651442 |
| chr_3 | 15481719 | 15961755 |
| chr_3 | 16281795 | 23322452 |
| chr_3 | 24072532 | 24712583 |
| chr_3 | 24912612 | 26132718 |
| chr_3 | 35673630 | 35863639 |
| chr_3 | 36223682 | 40124042 |
| chr_3 | 80807902 | 81767983 |
| chr_3 | 86408432 | 86998478 |
| chr_4 | 2592376 | 2882407 |
| chr_4 | 8163286 | 8323296 |
| chr_4 | 41668758 | 42018799 |
| chr_4 | 50590215 | 51530352 |
| chr_4 | 53770735 | 61822033 |
| chr_4 | 66582827 | 71213567 |
| chr_4 | 77044536 | 84685767 |
| chr_6 | 37029806 | 37479836 |
| chr_7 | 66698017 | 68448204 |
| chr_8 | 61787579 | 63567778 |
| chr_8 | 63867825 | 64077838 |
| chr_8 | 71788759 | 71958767 |
| chr_8 | 72158803 | 72688854 |
| chr_9 | 2181214 | 2611286 |
| chr_9 | 12883544 | 16584327 |
| chr_10 | 19173532 | 19373545 |
| chr_10 | 21613849 | 22013887 |
| chr_11 | 4180878 | 5170979 |
| chr_11 | 13531940 | 14041986 |
| chr_11 | 16452271 | 16702289 |
| chr_11 | 20232701 | 37754680 |
| chr_11 | 43125301 | 45075512 |
| chr_12 | 20432437 | 21662552 |
| chr_14 | 42952842 | 43072846 |
| chr_14 | 56685558 | 56845570 |
| chr_14 | 60756363 | 61016395 |
| chr_15 | 1680814 | 2010850 |
| chr_15 | 2430930 | 6051474 |
| chr_15 | 6291526 | 8151798 |
| chr_15 | 27974876 | 28464936 |
| chr_16 | 30574958 | 33395331 |
| chr_16 | 36235734 | 36625774 |
| chr_16 | 37145859 | 37935953 |
| chr_16 | 55858424 | 56348477 |
| chr_16 | 56628530 | 58618789 |
| chr_18 | 15764152 | 16774318 |
| chr_18 | 17494468 | 17614472 |
| chr_18 | 39828555 | 40468654 |
| chr_18 | 41248815 | 42399007 |
| chr_18 | 43369203 | 43499208 |
| chr_19 | 23135105 | 23235105 |
| chr_19 | 24035285 | 25145486 |
| chr_19 | 47029865 | 48540146 |
| chr_19 | 50450546 | 50600556 |
| chr_20 | 34158388 | 34468435 |
| chr_21 | 29905850 | 30165869 |
| chr_21 | 30775954 | 31225996 |
| chr_22 | 30492099 | 32212368 |
| chr_22 | 32522436 | 35492913 |
| chr_23 | 20086243 | 20466313 |
| chr_23 | 20796421 | 22906923 |
| chr_25 | 2981141 | 3451200 |
| chr_25 | 45487894 | 45807929 |
| chr_27 | 17282616 | 18282738 |
| chr_28 | 28968666 | 31309290 |
| chr_29 | 2401398 | 3651792 |
| chr_29 | 36553087 | 37683440 |
| chr_30 | 35552234 | 36892650 |
| chr_31 | 540962 | 5602782 |
| chr_31 | 6153020 | 6303039 |
| chr_31 | 14296006 | 14986223 |
| chr_31 | 16016637 | 16916931 |
| chr_31 | 24309678 | 25760173 |
| chr_31 | 29781685 | 30802022 |
| chr_32 | 22055208 | 22355267 |
| chr_33 | 27876080 | 28546194 |
| chr_34 | 1960929 | 3501275 |
| chr_34 | 3701347 | 3811350 |
| chr_34 | 4151455 | 4251455 |
| chr_34 | 5261722 | 5411734 |
| chr_36 | 13335435 | 13605484 |
| chr_37 | 6214780 | 13787247 |
| chr_X | 868199 | 1258224 |
| chr_X | 107967182 | 108437213 |

**Table 28.**

**Table 29.**

| **Gene** | **Isoform** | **Fragment id** | **Assay type** | **Forward primer id** | **Reverse primer id** | **Forward primer** | **Reverse primer** |
| --- | --- | --- | --- | --- | --- | --- | --- |
| NEBL | LIM-Nebulette | LIM_N_2 | Target | LIM_N_2_F | LIM_N_2_R | GGTGGCAGACACACCTGAA | CCTGCCTTTGCTTTCTTCAA |
| NEBL | LIM-Nebulette | LIM_N_3 | Target | LIM_N_3_F | LIM_N_3_R | AAATCTTCGCCTGAAACAGC | CTGCCTTTGCTTTCTTCAAA |
| NEBL | Nebulette | Nebulette_3 | Target | Nebulette_3_F | Nebulette_3_R | TCTGTGGCAGATACTCCTGAAA | GCTGTGCCAGCTCCTACTTC |
| NEBL | Nebulette | Nebulette_4 | Target | Nebulette_4_F | Nebulette_4_R | TCCTGAAATGGAAAGAGTGAAGA | CCTTTCATCTGTTTATGGTCCTG |
| MLLT10 | NA | MLLT10 | Target | MLLT10_1_F | MLLT10_1_R | GATGTCGACAGGCTTTCCAT | TGCACCATTACCTTCTTCTTCA |
| SKIDA1 | NA | SKIDA1_1 | Target | SKIDA1_1_F | SKIDA1_1_R | TGGGAAAGCGACCTGTACTT | TTTCAGGTGCTGCTGTTAGG |
| C10orf113 | NA | C10orf113 | Target | C10orf113_F | C10orf113_R | GAGTTTGTGTTTTACAAATCAGCTAGT | TCCATGCTTCCCTCTGGTTA |
| RPL13A | NA | RPL13A | Reference | RPL13A_F | RPL13A_R | ATGGGTCTTGAGGACCTCTG | AAGGCCAAGATCCATTATGC |
| RPL32 | NA | RPL32 | Reference | RPL32_F | RPL32_R | ATGCCCAACATTGGTTACGG | CTCTTTCCACGATGGCTTTG |
| G3P | NA | G3P | Reference | G3P_F | G3P_R | TGTCCCCACCCCCAATGTATC | CTCCGATGCCTGCTTCACTACCTT |

**Table 30.**

| **SNV id** | **Oligo type** | **Oligo id** | **Oligo sequence** |
| --- | --- | --- | --- |
| NEBL 1 | Reference forward | NEBL1_refF | TCTGGCACaattaaaaaattaatatttttCGCAGAAAGAGG |
| NEBL 1 | Reference reverse | NEBL1_refR | CCTCTTTCTGCGaaaaatattaattttttaattGTGCCAGA |
| NEBL 1 | Alternative forward | NEBL1_altF | TCTGGCACaattaaaaaattgatatttttCGCAGAAAGAGG |
| NEBL 1 | Alternative reverse | NEBL1_altR | CCTCTTTCTGCGaaaaatatcaattttttaattGTGCCAGA |
| NEBL 1 | Reference forward biotinylated | NEBL1_refFbio | /5Biosg/TCTGGCACaattaaaaaattaatatttttCGCAGAAAGAGG |
| NEBL 1 | Alternative forward biotinuylated | NEBL1_altFbio | /5Biosg/TCTGGCACaattaaaaaattgatatttttCGCAGAAAGAGG |
| NEBL 1.2 | Alternative forward NEBL 1.2 on alternative NEBL 1.1 background | NEBL1.2_D_altF | TCTGGCACaattaaaaaattgatatttttCACAGAAAGAGG |
| NEBL 1.2 | Alternative reverse NEBL 1.2 on alternative NEBL 1.1 background | NEBL1.2_D_altR | CCTCTTTCTGTGaaaaatatcaattttttaattGTGCCAGA |
| NEBL 1.2 | Alternative forward NEBL 1.2 on reference NEBL 1.1 background | NEBL1.2_S_altF | TCTGGCACaattaaaaaattaatatttttCACAGAAAGAGG |
| NEBL 1.2 | Alternative reverse NEBL 1.2 on reference NEBL 1.1 background | NEBL1.2_S_altR | CCTCTTTCTGTGaaaaatattaattttttaattGTGCCAGA |
| NEBL 1.2 | Alternative forward NEBL 1.2 on alternative NEBL 1.1 background, biotinylated | NEBL1.2_DFbio | /5Biosg/TCTGGCACaattaaaaaattgatatttttCACAGAAAGAGG |
| NEBL 1.2 | Alternative forward NEBL 1.2 on reference NEBL 1.1 background, biotinylated | NEBL1.2_SFbio | /5Biosg/TCTGGCACaattaaaaaattaatatttttCACAGAAAGAGG |
| NEBL 2 | Reference forward | NEBL7_refF | TGCCTGACAGCCCTGCCTGTCTGCTTTCATCATGGGTTTT |
| NEBL 2 | Reference reverse | NEBL7_refR | AAAACCCATGATGAAAGCAGACAGGCAGGGCTGTCAGGCA |
| NEBL 2 | Alternative forward | NEBL7_altF | TGCCTGACAGCCCTGCCTGTTTGCTTTCATCATGGGTTTT |
| NEBL 2 | Alternative reverse | NEBL7_altR | AAAACCCATGATGAAAGCAAACAGGCAGGGCTGTCAGGCA |
| NEBL 2 | Reference forward biotinylated | NEBL7_refFbio | /5Biosg/TGCCTGACAGCCCTGCCTGTCTGCTTTCATCATGGGTTTT |
| NEBL 2 | Alternative forward biotinuylated | NEBL7_altFbio | /5Biosg/TGCCTGACAGCCCTGCCTGTTTGCTTTCATCATGGGTTTT |
| NEBL 3 | Reference forward | NEBL2_refF | GCAGCAGACCCCAGCAGAGTGAAGAGTGTCGTGGGGACTT |
| NEBL 3 | Reference reverse | NEBL2_refR | AAGTCCCCACGACACTCTTCACTCTGCTGGGGTCTGCTGC |
| NEBL 3 | Alternative forward | NEBL2_altF | GCAGCAGACCCCAGCAGAGTAAAGAGTGTCGTGGGGACTT |
| NEBL 3 | Alternative reverse | NEBL2_altR | AAGTCCCCACGACACTCTTTACTCTGCTGGGGTCTGCTGC |
| NEBL 3 | Reference forward biotinylated | NEBL2_refFbio | /5Biosg/GCAGCAGACCCCAGCAGAGTGAAGAGTGTCGTGGGGACTT |
| NEBL 3 | Alternative forward biotinuylated | NEBL2_altFbio | /5Biosg/GCAGCAGACCCCAGCAGAGTAAAGAGTGTCGTGGGGACTT |
| NEBL 4 | Reference forward | NEBL4_refF | TCCCTAAATGAATTTAACAATTGCTGTGAACCTTTTTGTG |
| NEBL 4 | Reference reverse | NEBL4_refR | CACAAAAAGGTTCACAGCAATTGTTAAATTCATTTAGGGA |
| NEBL 4 | Alternative forward | NEBL4_altF | TCCCTAAATGAATTTAACAACTGCTGTGAACCTTTTTGTG |
| NEBL 4 | Alternative reverse | NEBL4_altR | CACAAAAAGGTTCACAGCAGTTGTTAAATTCATTTAGGGA |
| NEBL 4 | Reference forward biotinylated | NEBL4_refFbio | /5Biosg/TCCCTAAATGAATTTAACAATTGCTGTGAACCTTTTTGTG |
| NEBL 4 | Alternative forward biotinuylated | NEBL4_altFbio | /5Biosg/TCCCTAAATGAATTTAACAACTGCTGTGAACCTTTTTGTG |

**Table 31.**
