# Supplementary material for: The genetic consequences of dog breed formation—Accumulation of deleterious genetic variation and fixation of mutations associated with myxomatous mitral valve disease in cavalier King Charles spaniels
Source: PLoS Genet. 2021 Sep 2;17(9):e1009726. doi: 10.1371/journal.pgen.1009726 (PMC8412370; doi:10.1371/journal.pgen.1009726)
Supplement: Attachment — Submitted filename: Replies to reviewers_RESUBMITTED.docx [file pgen.1009726.s005.docx]

First, we would like to thank all reviewers for their insightful comments.

Below is an itemized list of replies to reviewer’s comments:


**Reviewer #1:**

**Comment 1. What are the genetic relationships of the eight breeds, do they belong to the same dog clade, to different clades? It would interesting to present this to readers for two reasons (1) general information about these 8 breeds and (2) do cavalier King Charles spaniels are outlier of all others or cluster within a subgroup?**

**Reply to comment 1:** We have added a phylogenetic tree (Fig 3 in supporting information) and a sentence in the Results section that refers to this tree (P. 10):

*“The breeds investigated here are approximately equally distantly related (Fig. 3 in Appendix S1), each representing a separate European breed clade, with the exception of golden retriever and Labrador that are more closely related, reflecting their common retriever origin (Parker et al., 2017).”*

**Comment 2. "To identify variants linked to this disease we next characterize mutations that are common in cKCs, but rare in other breeds, and then investigate if these mutations can predict MMVD in dachshunds". Why specifically dachshunds ? I understand because the risk alleles segregate at intermediate frequencies while seem to be fixed in cKCs ? Is that all ? Are there other breeds with this heart condition with good clinical data?**

**Reply to comment 2:** One other reason for selecting dachshund for this analysis is that MMVD has been shown to be heritable in this breed. We have updated the Methods section on P.43, L.16 to reflect this:

*“The Dachshund breed was selected for this purpose because, similar to in cKCs, MMVD is heritable in dachshunds (Olsen et al., 1999), but the prevalence of MMVD in this breed is moderate arguing that potential risk alleles that have become fixed in cKCs may be segregating and therefore detectable by association analysis in Dachshund.”*

Other breeds also develop MMVD as we describe in the introduction P.8, L.10, but good clinical data is probably not available for any breeds, but rather need to be collected similar to what we did for dachshunds here.

**Comment 3. "breed dogs carry 2-3% more derived, potentially deleterious, alleles at evolutionarily conserved amino acid changing sites than wolves". How is defined a derived allele ? I image several ways - postulate that ancestral allele is from a wild canid, from the wolf ? or using dog variant calling the REF allele is assumed as the ancestral allele and ALT allele the derived?. Well I think I understand the ALT allele is used when the authors use the statistic that measures the number of derived mutations observed in breed A that are not observed in breed B, and then calculates the ratio, RA/B, of the two numbers. Maybe the authors should provide some explanations of the derived allele, this could be clarified.**

**Reply to comment 3:** Yes, ancestral alleles are those that are observed in a not too distantly related outgroup (Andean fox). We explain how ancestral states are inferred in the Methods section P 38, L 7:

*“Publicly available whole genome sequence data from an Andean fox (Auton et al., 2013) was used to infer ancestral states of dog variants.”*

**Comment 4. "we calculated average pairwise FST for all variants across all breeds to identify high frequency derived alleles". Why not a across-breeds chi-sq test or fisher test ?**

**Reply to comment 4:** Chi-square and Fisher tests could have been used as well. We preferred to identify high frequency alleles in cKCs based on average pair-wise FST as this statistic readily identifies the breed in which the allele frequency shift has occurred and provides an intuitive measure of the extent of the allele frequency shift in that breed. Also, as we were primarily interested in identifying deleterious alleles associated with MMVD that may have drifted to high allele frequencies in cKCs we used a hard cut-off for average pairwise F_ST_ to identify the bulk of high frequency variants in cKCs, rather than Chi-square and Fisher tests to identify extreme outliers (which would have been more appropriate if our aim was to identify variants under e.g. selection).

**Comment 5. page 18 : therefor > therefore**

**Reply to comment 5:** Thank you! We have now corrected this.

**Comment 6. How do you understand that accelerated accumulation of mutational load during breed formation is seen only in cKCs at least for the additive model for allelic effects, ("cKCs carry 6.4% to 12.5% more derived mutations at highly conserved sites relative to the other breeds") ? No accelerated accumulation of mutational load is observed in other breeds because of different breeding practices ? because of demographic effect during/after breed formation ? and would that mean that the method to use mutational load to investigate a disease can not be transferred to many dog breeds ? to many diseases ?**

**Reply to comment 6:** We suggest two hypothesis to explain the additional mutational load observed in cKCs: 1) less efficient purifying selection in cKCs due to more, narrower and longer bottlenecks during cKCs breed formation relative to in other breeds or 2) hitchhiking of deleterious alleles in regions under artificial selection in cKCs. We attempted to test these hypotheses by 1) looking for a correlation in the relative amount of excess load and the difference in levels of genetic variation observed between breed pairs and 2) by filtering out regions that may have been under selection in cKCs. Our analyses however indicate that none of these hypotheses provide a sufficiently good answer. Based on the fact that our analyses, and those of previous authors, indicate that cKCs has experienced a particularly severe reduction in effective population size during breed formation we however speculate that hypothesis 1 still may be relevant and write like this in the Discussion section on P. 26:

*“the relative increase in deleterious variants in cKCs is almost constant (10-13% more derived alleles at the most conserved class of sites) irrespective of the breed used for comparison, i.e. there is no apparent correlation between relative amounts of mutational load and differences in overall levels of genetic variation between breeds, as may have been expected if mutational load accumulated as a consequence of less efficient selection in small populations. We have no simple explanation to this observation but speculate that the intensity and duration of the bottleneck(s) leading to breed formation may have been extreme in cKCs relative to in most other breeds.“*

We furthermore conclude on P. 28 in the Discussion:

*“…we find no simple relationship between overall levels of genetic variation and additive mutational load among the eight breeds analyzed here. Complex interplays between different modes of selection (dominant, additive, recessive), selection coefficients, hitchhiking, and extent and duration of bottleneck(s) may potentially modulate mutational load such that no simple relationships can be expected.”*

**Comment 7. It would be nice to discuss how to transfer this approach (genetic load in breeds assuming additive or recessive model for allelic effects), into a more general approach to investigate disease when using the dog model and WGS data.**

**Reply to comment 7:** It is not our ambition to use estimates of relative mutational load in dog breeds to help identify disease associated mutations. The two approaches - 1) estimating mutational load and 2) analyses of disease association - should be considered as separate, although conceptually linked analyses (increase in mutational load leads to more disease). The analysis of load is based on allele frequency shifts at many sites and unfortunately has little power to identify individual deleterious mutations that have increased in frequency.

**Comment 8. Dachshund association analysis. This paragraph (p18) is more about the statistics results for 10 selected candidate variants, rather than an association analysis. I suggest to change the paragraph title.**

**Reply to comment 8:** While we are not conducting a genome-wide associating analysis, we would argue that the correct term for a statistical analysis testing if one or more genotypes co-occur with a particular phenotype in a population is “association analysis”, and we would therefore prefer to keep this title.

**Comment 9. Across-breed association test: In this paragraph, you test if allele frequencies of the candidate variants can predict the incidence of MMVD. Is that done within each breed ? I suspect not. All breeds are used together for a linear regression test between genotype and the disease status ? Could you make that clearer.**

**Reply to comment 9:** Yes, that is correct, this test is not done within each breed, but across all breeds and uses breed-averages as variables. We have tried to clarify this in two sections of the manuscript:

The Results paragraph on P. 25 has been updated to make it clear that we are comparing breed-specific average allele frequencies with breed-specific MMVD incidence. It reads:

*“We then also genotyped the ten candidate variants (including the four nonsynonymous variants) in five additional Swedish dog populations (cocker spaniel, Norfolk terrier, Norwich terrier, Grand Danois and Dobermann pinscher) to investigate if breed-specific, average allele frequencies, at any of the candidate variants can predict breed-specific incidence of MMVD in a total set of 14 breeds (the five breeds mentioned above, the eight whole genome sequenced breeds and dachshunds).”*

Similarly the Methods section on P. 50 has been updated to make this more clear and now reads:

*“As a further means of exploring if any of the candidate MMVD variants identified in this study may affect the risk of developing MMVD in other breeds than dachshunds, we tested if average breed-specific allele frequencies at candidate variants could predict incidence of MMVD across a small set of dog breeds.”*

***Reviewer #2:*** 

**Comment 10. The only major thing is maybe, the authors have done extensive work and would be good make the programming scripts available, so with the raw sequence data made available, maybe some part of the work can be considered reproducible.**

**Reply to comment 10:** We have now made the scripts available on Github and updated the Methods section on P. 39 with a reference to the Github repository:

*“SNVs and indels were first annotated using SNPeff (Cingolani et al., 2012) and structural variants using custom written Perl scripts available at* [*https://github.com/erik-axelsson/delvar_MMVD/*](https://github.com/erik-axelsson/delvar_MMVD/) *(this repository contains all scripts used for all bioinformatic analyses described in this article).”*

**Comment 11. Sequencing and variant discovery  The authors need to explain what is FDR in the methods.**

**Reply to comment 11:** We have added this description of how FDR was calculated on P. 39 in the Methods section:

*“Power, accuracy and false discovery rate (FDR) of the SNV calling and genotyping was estimated by comparing variant calls from our whole genome resequencing data with those based on array genotyping (HD 170 canine array) in the same set of 20 cKCs individuals sequenced here (Vaysse et al., 2011). Power was calculated as the fraction of biallelic variable sites detected using array genotyping that was detected using whole genome resequencing. Similarly, genotype accuracy was estimated as the fraction of overlapping WGS calls showing identical genotypes in the array data. The FDR of the SNV discovery was estimated for sites interrogated using array genotyping (i.e. present on the HD 170 canine array) and calculated as the fraction of these sites that were variable in our WGS data that were found not to be variable using array genotyping.*

*”*

**Comment 12. MMVD coding candidate variants
The citation for genes with known function of potential relevance to MMVD pathology**

**Reply to comment 12:** We have added citations to the table legend for Table 2.

***Reviewer #3:*** 

**Comment 13. First of all a lot of information is included in this manuscript. I think the manuscript could be improved if it gets shortened. For example much of the information presented in the Introduction and Results are repeated in the Discussion and Methods. By vetting out the repetitions the manuscript would get easier to read.**

**Reply to comment 13:** We have removed or shortened redundant text throughout the manuscript. The length of the new manuscript remains almost identical to the first submitted version, despite many new text additions in response to other comments made by the reviewers. At some sections in the Results and Discussion we would however prefer to retain short reminders of reasons for an analysis or short reminding descriptions of methods for clarity and flow of the text. The following sections have been changed/shortened:

- Redundant comparison of our estimates of levels of genetic variation in dog breeds with previously published array based estimates removed from Results section (p. 10).

- Redundant description of the function of genes affected by non synonymous mutations in Results (p. 15-16) have been removed or moved to Discussion (p. 29).

- Redundant description of dachshund MMVD examination in Results section removed (p 18).

- Detailed and redundant description of origin of cell lines used for EMSA also present in Methods shortened in Results section (p. 22).

- Redundant description of Luciferase construct, also present in the Methods section, removed from Results (p. 23)

- Redundant description of MMVD incidence estimation method removed from Results (p. 25).

**14. A bit more background on dog domestication demographics would be good. Page 5 L9-19 mentions the reduction in Ne during domestication but just the fold reduction from wolf to dog. It would give the reader a better idea for the Ne for the breeds included in present study if you include the estimated values for each breed as well.**

**Reply to comment 14:** We have updated the description of the fold change in effective population size associated with domestication to now also include a specific example from the study referred to:

*P5. L9: “The initial phase of dog domestication, up until the start of recent breed formation, was associated with an estimated 16-fold reduction in effective population size (Ne) (from more than 32,000 to 1640-1980 individuals in the Basenji breed for instance), resulting in an Ne five times smaller than that of the extant wolf population (Freedman et al 2014).”*

Absolute population size estimates for all breeds in our study are however either not available from a single previous study or highly variable depending on what methodology the estimates are based on. We therefore do not refer to specific estimates of *Ne* for the breeds studied here but rather refer to previous analyses of levels, and patterns of genetic variation, which conclude that the demographic histories vary substantially between breeds and now also include a description of the range across which estimates of recent effective population sizes range across dog breeds in general:

*P5. L23: “Breed formation led to further reductions in Ne but the magnitude and duration of the breed bottlenecks varied substantially (Axelsson et al., 2012; Boyko et al., 2010). (Dreger et al (2016) noted that recent Ne for 80 analysed dog breeds span a five-fold range) arguing that different breeds may have continued to accumulate deleterious variants at different rates.”*

In the results section we compare several statistics to illustrate the potential differences in effective population size between the breeds analysed in the manuscript and now also included a new site frequency-based modelling of the demographic history of each breed (Fig. 5 in Appendix).

**15. It’s mentioned several times that the cKCs have been through a more intense bottleneck(s) than the other breeds. Please include a reference for this or show it with your results i.e., heterozygosity, ROH. It also makes the reader wonder if some of the breeds have been through consecutive bottlenecks. Given the large literature on dog domestication this information is likely obtainable.**

**Reply to comment 15:** We have rephrased a section of the results (P. 10) where we describe that cKCs, relative to the other breeds, have fewer segregating sites, is less heterozygous, have longer LD and more homozygous regions. The section reads:

*“Congruent with expectations from the multiple bottlenecks believed to be associated with dog domestication and breed formation, we observe an excess of high frequency derived variants and a lack of rare variants throughout all breeds analysed here (Fig. 4 in Appendix S1) and formal site frequency based demographic modelling suggests that most breeds have undergone at least two bottlenecks (Fig. 5 in Appendix S1). Levels of genetic variation however vary substantially between breeds, with relatively high levels in beagles and Labradors, moderate levels in standard poodles, German shepherds and golden retrievers, and low levels in West Highland white terriers (WHwt) and Rottweilers, indicating that the duration or intensities of bottlenecks varied significantly across breeds (Table 1). cKCs carry significantly fewer segregating sites (Table 1) and is less heterozygous (Table 2 in Appendix S1) than any of the other breeds, a pattern that is consistent across all categories of variants (Table 1). In line with a more extreme bottleneck during the history of cKCs, linkage disequilibrium extends over longer distances (Fig. 6 in Appendix S1) and more regions are homozygous (Fig. 7 in Appendix S1) in cKCs relative to in other breeds.”*

We have also included a new site frequency-based modelling of past population size changes in the breeds analysed here. This analysis infers that two bottlenecks (one early, probably at initial domestication and one more recent, potentially associated with breed formation) affected most breeds during dog domestication, but indicate that at least the most recent of these bottlenecks were particularly narrow in cKCs. We discuss this new analysis briefly on page 27 in the Discussion:

*“Likewise, site frequency-based demographic inferences suggest that the more recent of the two inferred bottlenecks associated with dog domestication may have been particularly narrow in cKCs (Fig. 5 in Appendix).”*

On page 27 in the Discussion we also describe historical evidence of what likely constitute multiple recent breed bottlenecks in cKCs:

*“Historical records suggest that small spaniel-type dogs have existed for at least 1,000 years and hint at a history involving serial bottlenecks. Small spaniels were popular at royal courts throughout Europe and Asia for several hundred years (Willes, Renee, 2013) and the appearance of cKCs in particular is very similar to toy spaniels kept by the royal Stuart family (rulers of Scotland, England and Ireland), including King Charles II (1630-1685). Over time a brachycephalic variety of these spaniels became the dominating type, but eventually the old, more long-nosed type was restored by a small breeding initiative starting in the 1920’s. This rescued variety however suffered a serious decline in numbers during the second world war before becoming recognized as a breed of its own (cKCs) in 1945.”*

**16. In the Discussion the authors speculate on the effect of past bottlenecks on the cKC breed (P27 L13-15). This could potentially be resolved by running some more demographic analyses.”**

**Reply to comment 16:** As described in our answer to comment 15 we now include a new site frequency-based inference of past population size changes in the breeds analysed here (Fig. 5 in Appendix).

**17. There seem to be two different stories merged into one. First one is the cKCs part and second the Dachshund part. It makes sense to include them both as the authors are searching for variants associated with MMVD, but I suggest that the authors make a better bridge between the two parts. For example flesh out more clearly why Dachshunds were chosen (I guess because the dataset were available?). Maybe you could move the sentence “The Dachshund breed was….” (P43 L6-9) to the Introduction?**

**Reply to comment 17:** We have reformulated the introduction of the dachshund in the Introduction section on p. 8:

*“To further assess whether any of these variants affect disease susceptibility in dogs, we screened a second breed that is also affected by MMVD - dachshund - for clinical and echocardiographic signs of the disease, and tested if candidate variant genotypes could be used to predict a diagnosis of MMVD.”*

We have also reformulated the motivation for choosing dachshund in the Results section in p. 18:

*“The Dachshund breed was selected for this purpose because, similar to in cKCs, MMVD is heritable in dachshunds (Olsen et al., 1999), but the prevalence of MMVD in this breed is moderate arguing that potential risk alleles that have become fixed in cKCs may be segregating and therefore detectable by association analysis in Dachshund.”*

*Minor comments*

**18. P9 L4: Is the dog genome chromosome level?**

**Reply to comment 18:** CanFam3.1 is not chromosome level, however the recently released CanFam4 is close to. Still this analysis was performed on CanFam3.1.

**19. P37 L24: the GATK settings for the different parameters are missing.**

**Reply to comment 19:** The statements regarding GATK parameters describes which parameters (these are not quantitative parameters) that were used for variant quality score calibration. All additional parameter settings are now also available in the publicly available code used for this project (see below).

**20. L6: GATK parameters not provided.**

**Reply to comment 20:** See reply to comment 19.

**21. P39 L3-10: This power analysis is a good inclusion.**

**Reply to comment 21:** Thanks!

**22. P39 L14: will these custom scripts be made available?**

**Reply to comment 22:** We have now made custom scripts available on Github. We refer to the Github site in the Methods section on p. 39:

*“SNVs and indels were first annotated using SNPeff (Cingolani et al., 2012) and structural variants using custom written Perl scripts available at* [*https://github.com/erik-axelsson/delvar_MMVD/*](https://github.com/erik-axelsson/delvar_MMVD/) *(this repository contains all scripts used for all bioinformatic analyses described in this article).”*

**23. P41 L18: Is LD information available for canines?**

**Reply to comment 23:** Yes, LD has been estimated for many breeds previously and we refer to one of these studies (Boyko et al 2010) in this paragraph in the Discussion on p25:

*“This hypothesis is consistent with previous array-based analyses in which cKCs rank among the ~10 least variable breeds out of 80 analysed (Boyko et al., 2010; Parker et al., 2017) and combinations of both long-range linkage disequilibrium and extensive runs of homozygosity in cKCs indicate that both older population contractions and recent inbreeding may indeed have been pronounced (Fig. 6 and 7 in Appendix)(Boyko et al., 2010).”*

**24. P44 L23-25: age, sex and genotype is included in the linear regression. I wonder if other traits like body mass, fitness etc. could influence the onset of MMVD, and if so the authors also tested these in the linear reg?**

**Reply to comment 24:** Body weight does not correlate with severity of MMVD signs within dachshunds **(***J Vet Intern Med* 1999;13:448–456 ) and was hence not modelled in our linear regression. Fitness was also not modelled as, to our knowledge, there are no studies indicating that fitness affects age at onset for MMVD. In addition, data on fitness is not available.

**25. P45 L18-19: This sounds like it’s a laboratory line of dogs. If so is it selected to have higher expression of MMVD?**

**Reply to comment 25:** It is a population of beagles mostly used for educational purposes at the Swedish Agricultural University and it has not been intentionally bred for high MMVD incidence.

**26. Figure 2e: it looks like a label is missing on this figure. The left part is an undescribed smear.**

**Reply to comment 26:** We have added a label for the EBNA control lanes of the electrophoretic mobility shift assay depicted in figure 2e. The smear resulted from failure to properly attach the membrane to the gel at that particular spot, hence there's no imprint in that part. The spot does however not interfere with the readouts of neither the EBNA control, nor the probes assayed.
